# Integration of Scales and Cameras in Nondisruptive Electronic Beehive Monitoring: On the Within-Day Relationship of Hive Weight and Traffic in Honeybee (*Apis mellifera*) Colonies in Langstroth Hives in Tucson, Arizona, USA

**DOI:** 10.3390/s22134824

**Published:** 2022-06-25

**Authors:** Vladimir Kulyukin, Anastasiia Tkachenko, Kristoffer Price, William Meikle, Milagra Weiss

**Affiliations:** 1 Department of Computer Science, Utah State University, Logan, UT 84322, USA; anastasiia.tkachenko@usu.edu (A.T.); kristoffer.price@usu.edu (K.P.); 2 Carl Hayden Bee Research Center, USDA-ARS, Tucson, AZ 85719, USA; william.meikle@usda.gov (W.M.); milagra.weiss@usda.gov (M.W.)

**Keywords:** electronic beehive monitoring, continuous beehive monitoring, digital apiculture, bee traffic, hive weight, physical sensors, sensor devices, sensor systems, *Apis mellifera*, correlation, chi-square

## Abstract

The relationship between beehive weight and traffic is a fundamental open research problem for electronic beehive monitoring and digital apiculture, because weight and traffic affect many aspects of honeybee (*Apis mellifera*) colony dynamics. An investigation of this relationship was conducted with a nondisruptive two-sensor (scale and camera) system on the weight and video data collected on six *Apis mellifera* colonies in Langstroth hives at the USDA-ARS Carl Hayden Bee Research Center in Tucson, Arizona, USA, from 15 May to 15 August 2021. Three hives had positive and two hives had negative correlations between weight and traffic. In one hive, weight and traffic were uncorrelated. The strength of the correlation between weight and traffic was stronger for longer time intervals. The traffic spread and mean, when taken separately, did not affect the correlation between weight and traffic more significantly than the exact traffic counts from videos. Lateral traffic did not have a significant impact on weight.

## 1. Introduction

Hive weight is an important indicator of colony activity [1], and many amateur and commercial operations measure weight continuously to estimate colony food reserves and to gauge optimal honey harvesting times [2,3]. Weight changes are indicative of the forager loss and gain during the day and of pollination activity [4,5]. Traffic is another important factor affecting colony dynamics. Traffic at hive entrance may predict honey weight gain [6], and rapid traffic increases at hive entrance may be due to robbing and swarming events [7]. Continuous video traffic measurement is a nondisruptive, robust, and inexpensive method to estimate hive traffic levels [8]. Sensor-based methods of monitoring colonies have shown their effectiveness in estimating the effects of stressors (e.g., poor nutrition or agrochemical exposure) on colony foraging activity and thermoregulation that are difficult to detect using other means such as visual colony assessments by human beekeepers [9]. Electronic beehive monitoring researchers have used sensors to measure internal and external temperature, humidity, atmospheric pressure, wind direction and speed, rainfall, shortwave radiation, weight, and traffic. While many researchers have investigated the relationship between traffic and weather (e.g., [10,11,12]) or weight and weather (e.g., [9,13,14,15,16]), the literature on continuous beehive monitoring, with few notable exceptions (e.g., [6]), has a dearth of studies on the relationship of hive weight and traffic. This problem is fundamental, because hive weight and traffic affect many aspects of colony dynamics. Furthermore, hive weight is a function of several factors such as colony food collection and consumption, bee development and loss, moisture gain or loss due to nectar inflow, ambient humidity and bee respiration, water inflow and outflow, robbing and swarming, and external weather events. Some of these factors are associated with colony traffic, while others are not. For example, humid weather adds to hive weight, because moist wood (and many hives worldwide are made out of wood) is heavier than dry wood. Thus, the weight change due to humidity, especially at night when bees do not fly, is not associated with colony traffic. Thus, if we understand the relationship between hive weight and traffic, we can use both measurements more precisely to identify behavioral markers of *Apis mellifera* colonies and to improve data interpretation.

The study by Marceau et al. [6] is a rare attempt to shed light on the relationship between the traffic at hive entrance estimated with an electronic bee counter and hive weight on five Langstroth hives with *Apis mellifera* at an apiary of 22 Langstroth hives for 35 days in July and August 50 km west of Quebec city. The monitored period of each day was from 9:00 to 16:00. For the first four hives, traffic counts were recorded at 16:00 for the 9:00–16:00 period, and the weight difference for every 24 h period was logged at 9:00. The fifth hive was automatically monitored at 15 min intervals using data logging equipment from 9:00 to 16:00 for the mean daily traffic activity (bees/h), and the hive weight difference (kg) was recorded for the 24 h period at 9:00. The researchers proposed the quadratic model $GAIN=B_{0}+B_{1}\cdot ACT^{2}$, where GAIN estimates the hive honey gain (kg), ACT is the average bee activity between 9:00 and 16:00 (bees/h), and $B_{0}$ and $B_{1}$ are model coefficients. Marceau et al. reported that the honey gain varied from 28.7 to 58.4 kg and that the average bee activity for the 35 observation days varied from 19,403 bees/h for the least productive hive to 27,408 bees/h for the most productive hive. The four resulting models were very similar, with the best curve fitting obtained on the two most productive hives with $R^{2}=0.88$ and $R^{2}=0.90$. The researchers concluded that the more active a colony was, the more honey it produced, and the minimum activity rate required to obtain a positive daily gain was 14,000 bees/h. When the daily average activity remained below 14,000 bees/h, the hive weight decreased. While the findings by Marceau et al. are significant, their investigation had several important limitations. First, hive weight can be only an approximate estimate of honey gain, because the latter is included in the former. Second, the directionality of bee motion was not taken into account. Specifically, traffic in the vicinity of the hive consists of incoming bees, outgoing bees, and laterally flying bees, which Marceau et al. did not take into account. Third, the researchers made no attempt to distinguish the weight associated with traffic and the weight not associated with it. Fourth, the researchers did not justify why traffic at hive entrance was estimated from 9:00 to 16:00. Research (e.g., [9]) shows that foragers start flying out as early as 5:00 and return to the hive as late 20:30 or even later. Fifth, the datasets described in the article do not appear to be publicly available for replication, standardization, and improvement.

Our investigation addresses the gap in the literature on the relationship of hive weight and colony traffic by investigating the within-day relationship between hive weight and traffic in the vicinity of a hive with a nondisruptive two-sensor (scale and camera) electronic beehive monitoring (EBM) system. We make the following contributions to the body of research on continuous hive monitoring. First, we formulate, prove, and experimentally validate a necessary condition for the *within-day* independence of weight and traffic on time periods from 1 h up to 6 h. Second, our experiments indicate that the correlation of weight and traffic becomes stronger on time periods longer than 1 h. Third, while the necessary condition for the independence was experimentally verified in our investigation, the executed $\chi^{2}$ tests failed to verify the implied sufficiency condition for the within-day independence of weight and traffic for any tested time period from 1 h up to 6 h. Thus, the formulation of the within-day sufficiency conditions remains an open problem for electronic beehive monitoring and theoretical apiary science. Fourth, our experiments show that some hives had positive and some hives had negative correlations between weight and traffic. We offer several conjectures on possible causes that may warrant further investigation. Fifth, the computed correlation coefficients and the executed $\chi^{2}$ tests showed that lateral traffic did not have a significant impact on weight change and may be omitted in within-day computational models that predict hive weight from traffic. Sixth, our experiments suggest that the traffic spreads and means, when taken separately, did not affect the correlation of weight and traffic more significantly than the exact traffic counts. Thus, exact traffic counts may suffice as traffic estimates. Finally, we made public our curated datasets of time-aligned weight and traffic measures from our field deployment at the USDA-ARS Carl Hayden Bee Research Center in Tucson, Arizona (AZ), USA, in May–August 2021. These datasets can be used as benchmarks for replication, standardization, and improvement.

Since EBM is a relatively recent branch of digital apiculture and does not yet have standard terminology, we conclude the introduction with several definitions. We use the terms *bee* and *honeybee* to refer to the *Apis mellifera* honeybee. We use the terms *hive* and *beehive* to refer to a standard Langstroth hive or a variant thereof with an *Apis mellifera* colony. We define the *vicinity* of a hive to be the cube-shaped space in front of the hive’s entrance with dimensions 3 m × 3 m × 3 m continuously monitored with a camera-computer unit. We use the term *traffic* to refer to all bee traffic in the vicinity of the hive. We use the term *total traffic* to refer to the bee traffic that includes *incoming traffic* (number of bees flying into the hive), *outgoing traffic* (number of bees flying out of the hive), and *lateral traffic* (number of bees flying parallel to the landing pad of the hive) over a given period of time. We use the term *electronic beehive monitoring* (EBM) to refer to the acquisition and analysis of digital data on the behavior of a managed bee colony through various sensors deployed in or around the hive. We use the adjectives *nondisruptive* and *noninvasive* with respect to EBM to describe the type of EBM that requires no structural modification of the hive and no deployment of active or passive sensors inside the hive or on individual bees. EBM solutions are nondisruptive insomuch as they do not disrupt any natural cycles of the monitored colonies and preserve the sacredness of the honeybee space. We note that, unlike other state-of-the-art EBM investigations (e.g., [5,16]) that rely on disruptive solutions (e.g., radio tags on bees or structural hive modifications), we used only nondisruptive methods in our study.

The remainder of our article is organized as follows. In Section 2, we detail the materials and methods of our investigation. In Section 3, we present our results. In Section 4, we discuss our results. In Section 5, we present our conclusions. Our supplementary materials include not only the datasets and additional tables and plots but also several short videos that illustrate important hardware and software aspects of our EBM system, which the readers may want to watch before proceeding to the remainder of the article. References to the figures and tables in the supplementary materials start with the prefix S (e.g., S54).

## 2. Materials and Methods

### 2.1. Data

The dataset was acquired during the deployment of 10 BeePi monitors (e.g., [17]) on *Apis mellifera* colonies in Langstroth hives at the USDA-ARS Carl Hayden Bee Research Center in Tucson, Arizona (AZ), USA (GPS coordinates: 32·13${}^{\prime }$18.274${}^{\prime \prime }$ N, 110·55${}^{\prime }$35.324${}^{\prime \prime }$ W) from 20 May to 15 August 2021. All colonies had Italian queens from two breeders: one in California and one in Hawaii. The queens were all painted (blue for breeder 1; green and yellow for breeder 2) to enable queen verification throughout the experiment. All queens were one year old. No hives swarmed during the monitored period. Each BeePi monitor was equipped with a Raspberry Pi 3 model B v1.2 computer coupled to a Raspberry Pi v2 8-megapixel camera. Timestamped 30-second mp4 25 frames per second videos were taken by each monitor every 15 min of the 3 m × 3 m × 3 m cube-shaped space in front of the hive on top of which the monitor was mounted. The videos were captured from 7:00 to 20:30 due to the poor visibility at the site apiary before 7:00 and after 20:30. The videos were saved on each monitor’s 5 TB USB storage device. All 10 hives were each placed on the 10 stainless steel electronic scales (Tekfa model B-2418 and Avery Weigh-Tronix model BSAO1824-200; max. capacity: 100 kg, precision: ±20 g; operating temperature: −30 °C to 70 °C) and linked to 16-bit dataloggers (Hobo UX120-006M External Channel data logger, Onset Computer Corporation, Bourne, MA). The hive weight was measured in kilograms (kg) and logged every 5 min, which was the default time period of the data logger. Four of the ten BeePi monitors were damaged during a severe storm in Tucson, AZ, in July 2021 and were fixed in early August 2021. However, due to this data acquisition gap, the data from these hives were not used in this investigation. We henceforth refer to the remaining six hives from which the weight and traffic data were collected by their IDs used in our logs: H17, H19, H41, H43, H47, and H53. Each of the 13,353 videos (see Table 1) collected from the six hives was processed by the BeePIV algorithm [8]. BeePIV converts video frames to particle motion frames, computes particle displacement vector fields, classifies individual displacement vectors as incoming, outgoing, and lateral, and uses vector counts (non-negative integers) to measure incoming, outgoing, and lateral bee traffic. Total traffic is estimated as the sum of the incoming, outgoing, and lateral measurements. The timestamps on weight and traffic measurements were used to time-align them into one CSV file for each hive. The final dataset consisted of six CSV files (one per hive) of time-aligned incoming (IN), outgoing (OUT), lateral (LAT), total (TOT) counts and weight measurements. Since weight measurements were logged every 5 min while traffic measurements were logged every 15 min, each weight measurement time-aligned with a traffic measurement was computed as the mean of the three weight measurements the middle of which had the same timestamp with the traffic measurements. Weight measurements were raw in that they included external impacts (e.g., someone puts a heavy object such as a brick or a super on top of the hive). Thus, when the weight rose or dropped abruptly by ≈20 kg and returned to the previous level within 30 min, which is physically impossible in a real beehive, the measurement was considered to reflect an external impact and was replaced with the mean of the neighbors before and after it. Table 1 summarizes the information on the CSV data files.

### 2.2. Hive Inspection and Treatment

All monitored hives had regular hive inspections carried out by the fifth author for the duration of the experiment. We counted frames of bees and mite drops and logged qualitative brood assessments. Frames of bees are counts of individual frames in a hive that are completely covered by bees on both sides. If only one side of a frame is completely covered with bees, then it is counted as 1/2 of a full frame. It should be noted that such measurements as 1/2, 1/4, 1/8 of a full frame are visual assessments by the beekeeper. Counts of frames of bees are an estimate of the overall health of a colony. Mite drops are counts of Varroa mites on a sticky board. A sticky board is a thin (≈2 cm thick) rectangular piece of corrugated plastic on which a thin film of Vaseline (or other adhesive substances such as plant oil) is placed with a paper towel. The sticky board was inserted into the screened bottom board underneath each monitored hive. As mites drop from bees in a colony, they stick to the board and can be visually counted by the beekeeper. Greater mite drops indicate higher levels of mite infestation, which may negatively impact the colony’s productivity. Brood assessments are the beekeeper’s qualitative assessments of the brood’s condition. We used the following qualitative labels in our brood assessments: straight, straight with punctured caps, spotty, PMS (parasitic mite syndrome), no brood, and chalk brood. Straight brood indicates a productive laying queen. Straight brood with punctured caps also indicates a productive queen with potential minor laying problems. Spotty brood shows that a queen lays eggs in isolated and typically disconnected cell regions, which may cause productivity problems or colony failure later on. PMS characterizes the brood with white larvae that appear chewed or sunken on the side of some cells. Chalk brood is caused by a fungus called *Ascosphaera apis*. Frames affected by chalk brood have white chunks of mummified brood that resemble small pieces of white chalk. This disease infects a hive through reproductive spores attached to pollen, robbing bees, or tools used in already infected hives. Hives infected with chalk brood often fail and present a danger to the other hives in the apiary due to bee drift. Apivar strips were applied to all monitored hives on 8 July 2021 to treat Varroa mites. We assessed the population strength of each colony by calculating the weight of the adult bee mass (bee mass) by subtracting from the total weight of the hive the combined weight of the woodenware, the electronics, and the frames without the bees [9]. The adult bee mass measurements were conducted twice on each hive at the beginning (June 2021) and at the end (August 2021) of the monitored period.

### 2.3. Random Variables and Correlations

We measured hive weight and traffic as two jointly observed random variables *W* (weight) and *T* (traffic). Our samples were

$$\left(W_{1},T_{1}\right),\ldots ,\left(W_{n},T_{n}\right),$$

where $W_{i}$ and $T_{i}$, 1≤i≤n, are time-aligned weight and traffic measurements for a given hive (see Table 1 for specific values of *n*). We use the notation $W_{t}$ and $T_{t}$ to denote random variables whose values range over the values of *W* and *T* at time *t*. The correlation between two random variables is typically measured with Pearson’s, Spearman’s, and Kendall’s correlation coefficients [18,19,20], denoted as $\rho_{P}$, $\rho_{S}$, and $\rho_{K}$, respectively. We tested the absence against the presence of correlation with the following hypotheses:

(1) $$\begin{array}{ccc} H_{\rho ,0}:\rho & = & 0;\\ H_{\rho ,1}:\rho & \neq & 0. \end{array}$$

where $\rho =\rho_{P}$, $\rho =\rho_{S}$, or $\rho =\rho_{K}$. $H_{\rho ,0}$ was rejected in favor of $H_{\rho ,1}$ at p≤0.05. We use the notation ${\hat{\rho }}_{P}$, ${\hat{\rho }}_{S}$, and ${\hat{\rho }}_{K}$ to denote the computed estimates of $\rho_{P}$, $\rho_{S}$, and $\rho_{K}$. Thus, we computed Pearson, a statistical measure of linear dependency between two variables, of *n* measurements $\left(W_{i},T_{i}\right)$ as

(2) $$\begin{array}{ccc} {\hat{\rho }}_{P} & = & \frac{\sum_{i=1}^{n}\left(W_{i}-\bar{W}\right)\left(T_{i}-\bar{T}\right)}{\sqrt{\sum_{i=1}^{n}{\left(W_{i}-\bar{W}\right)}^{2}}\sqrt{\sum_{i=1}^{n}{\left(T_{i}-\bar{T}\right)}^{2}}}. \end{array}$$

Spearman and Kendall measure a monotonic association between two random variables, and are more robust versions of Pearson, because they rely on ranks. The rank of an observation $W_{i}$, $R(W_{i})$, is its position in a list of measurements for a random variable sorted in ascending order. For example, if there are four measurements $W_{1}=6.3$, $W_{2}=8$, $W_{3}=2.5$, and $W_{4}=7.4$, then the sorted list is $\left(W_{3}=2.5,W_{1}=6.3,W_{4}=7.4,W_{2}=8\right)$ and the ranks are $R(W_{1})=2$, $R(W_{2})=4$, $R(W_{3})=1$, and $R(W_{4})=3$. We computed Spearman and Kendall of *n* time-aligned measurements $\left(W_{i},T_{i}\right)$ as

(3) $$\begin{array}{ccc} {\hat{\rho }}_{S} & = & \frac{\frac{1}{n}\sum_{i=1}^{n}R\left(W_{i}\right)R\left(T_{i}\right)-{\left(n+1\right)}^{2}/4}{(n^{2}-1)/12};\\ {\hat{\rho }}_{K} & = & \frac{\sum_{i=1}^{n}\sum_{j>i}^{n}I\left\{W_{i}≶W_{j},T_{i}≶T_{j}\right\}-\sum_{i=1}^{n}\sum_{j>i}^{n}I\left\{W_{i}≶W_{j},T_{i}≷T_{j}\right\}}{n(n-1)/2}, \end{array}$$

where the event $\left\{W_{i}≶W_{j},T_{i}≶T_{j}\right\}$ refers to the situation when the comparison signs between $W_{i}$ and $W_{j}$ and $T_{i}$ and $T_{j}$ are the same. Thus, if $W_{i}<W_{j}$, then $T_{i}<T_{j}$, and if $W_{i}>W_{j}$, then $T_{i}>T_{j}$. The event $\left\{W_{i}≶W_{j},T_{i}≷T_{j}\right\}$ refers to the situation when the comparison signs between $W_{i}$ and $W_{j}$ and $T_{i}$ and $T_{j}$ are different. Thus, if $W_{i}>W_{j}$, then $T_{i}<T_{j}$, and if $W_{i}<W_{j}$, then $T_{i}>T_{j}$.

We computed the correlation coefficient heat map of weight (W) and the five traffic types for each hive: IN, OUT, difference between IN and OUT (IN-OUT), sum of IN and OUT (IN+OUT), and total (TOT=IN+OUT+LAT). We used the autocorrelation function (ACF) to detect non-randomness in weight and traffic viewed as time series [21]. The ACF evaluates the similarity between a time series (i.e., the signal) and its copy with a shift, which is referred to as a lag. The ACF is a function of a lag. Let $S_{1},S_{2},\ldots ,S_{n}$ be a time series of observations, then the ACF of the lag *L* is defined as the Pearson correlation $\rho_{P}$ between $S_{i}$ and $S_{i+L}$ as

(4) $${\hat{\rho }}_{P}=\frac{\sum_{i=1}^{n-L}\left(S_{i}-\bar{S_{1:(n-L)}}\right)\left(S_{i+L}-\bar{S_{L:n}}\right)}{\sqrt{\sum_{i=1}^{n}{\left(S_{i}-\bar{S_{1:(n-L)}}\right)}^{2}}\sqrt{\sum_{i=1}{\left(S_{i+L}-\bar{S_{L:n}}\right)}^{2}}},$$

where $\bar{S_{1:(n-L)}}$ and $\bar{S_{1:(n-L)}}$ are the mean values of the series $S_{1},S_{2},\ldots ,S_{n-L}$ and $S_{L},S_{2},\ldots ,$$S_{n}$, respectively. Autocorrelation plots are used to visually assess the presence of trends and cycles in data. A trend is a pattern in a time series that does not repeat at least within the captured period. Cyclicity is a component that regularly repeats itself over time. If a time series has a trend, the ACF does not reach zero unless the lag is sufficiently long. If a time series contains a significant cycle, the autocorrelation plot typically shows spikes at multiples of lags equal to the period.

### 2.4. Weight and Traffic Changes

Since each BeePi monitor captured a 30-s video every 15 min from which the BeePIV algorithm extracted the integers IN, OUT, LAT, and TOT, we measured the change in traffic over a *lag* of 15k minutes as

(5) $$\begin{array}{ccc} \Delta_{k}T_{t} & = & \left|\sum_{i=1+(t-1)k}^{tk}T_{i}-\sum_{i=1+(t-2)k}^{(t-1)k}T_{i}\right|. \end{array}$$

Thus, if k=4, then the lag is 1 h; if k=8, then the lag is 2 h, etc. The values of $\Delta_{k}T_{t}$ were logarithmically transformed to make the rate of change distribution closer to normal as they approximately follow a log-normal distribution (see Figures S1 and S2 in the Supplementary Materials). Since weight measurements were logged every 5 min, for every $T_{i}$ the time-aligned $W_{i}$ was computed as the mean of $W_{i-1}$, $W_{i}$, and $W_{i+1}$. The change in weight over a lag of 15k minutes was computed as

(6) $$\begin{array}{ccc} \Delta_{k}W_{t} & = & |W_{1+tk}-W_{1+(t-1)k}|, \end{array}$$

where the values of 1+tk and 1+(t−1)k were chosen so that $W_{1+tk}$ and $W_{1+(t-1)k}$ always belonged to the video monitoring period (7:00–20:30) of the same day. The variables $\Delta_{k}W_{t}$ were assumed to be independent and identically distributed for any *k*. Specifically, if $k\neq k^{\prime }$, then $\Delta_{k}W_{t}$ and $\Delta_{k^{\prime }}W_{t}$ may *not* have identical distributions. However, if *d* and $d^{\prime }$ are two different days, $\Delta_{k}W_{t}$ on day *d* is assumed to be independent of $\Delta_{k}W_{t}$ on day $d^{\prime }$, but the distribution of $\Delta_{k}W_{t}$ is assumed to be identical on *d* and $d^{\prime }$. The variables $\Delta_{k}T_{t}$ were analogously assumed to be independent and identically distributed. We denote the weight change over the lag 15k as $\Delta_{k}W$ and the traffic change over the same lag as $\Delta_{k}T$. The change in the variance ($\sigma^{2}$) and the mean (μ) of *T* were computed as

(7) $$\begin{array}{ccc} \Delta_{k}\sigma^{2}{\left(T\right)}_{t} & = & |\sigma^{2}\left(T_{1+(t-1)k},T_{2+(t-1)k},...,T_{tk}\right)-\sigma^{2}\left(T_{1+(t-2)k},...,T_{(t-1)k}\right)|;\\ \Delta_{k}\mu {\left(T\right)}_{t} & = & |\mu \left(T_{1+(t-1)k},T_{2+(t-1)k},...,T_{tk}\right)-\mu \left(T_{1+(t-2)k},...,T_{(t-1)k}\right)|. \end{array}$$

An example of calculating $\Delta_{k}W$, $\Delta_{k}T$, $\Delta_{k}\sigma^{2}{\left(T\right)}_{t}$, and $\Delta_{k}\mu {\left(T\right)}_{t}$ is given in Appendix A.

### 2.5. Joint Probabilities

We defined the function

(8) $$D_{\epsilon }\left(Z_{t}\right)=I_{\epsilon }\left(\Delta_{k}Z_{t}\right)=\left\{\begin{array}{cc} 0, & if\;|Z_{1+tk}-Z_{1+(t-1)k}|<\epsilon \\ 1, & otherwise, \end{array}\right.$$

to be indicative of the event $\left\{\Delta_{k}Z_{t}=|Z_{1+tk}-Z_{1+(t-1)k}|\geq \epsilon \right\}$. The function divides the values of $\Delta_{k}Z_{t}$ into two categories: 0 and 1. Thus, if $\epsilon_{W}$ and $\epsilon_{T}$ are two thresholds for the change in weight and traffic, respectively, then $D_{\epsilon_{W}}\left(W_{t}\right)=1$ signifies the change in weight between times t−1 and *t* at or above $\epsilon_{W}$, while $D_{\epsilon_{T}}\left(T_{t}\right)=1$ signifies the change in bee traffic between t−1 and *t* at or above $\epsilon_{T}$. If ${\hat{Z}}_{i}$ is the estimate of $Z_{t}$ at time *i* (i.e., the observed value), the probability of $\left\{D_{\epsilon }\left(Z_{t}\right)=1\right\}$ can be estimated as the average of the occurrences of this event in *n* trials as

(9) $$P\left(D_{\epsilon }\left(Z_{t}\right)=1\right)\approx \frac{\sum_{i=1}^{n}D_{\epsilon }\left({\hat{Z}}_{i}\right)}{n}=P^{*}\left(D_{\epsilon }\left(Z_{t}\right)=1\right).$$

A necessary condition of the independence between the random variables of $X_{t}$, $Y_{t}$, $X_{t-1}$, and $Y_{t-1}$ can be formulated as follows and proved as a theorem (a proof is in Appendix A).

**A Necessary Condition for Independence (NCI):** If $X_{t}$, $X_{t-1}$, $Y_{t}$, $Y_{t-1}$ are discrete independent random variables, then, for any $\epsilon_{X}$ and $\epsilon_{Y}$,

(10) $$P\left(D_{\epsilon_{X}}\left(X_{t}\right)=1,D_{\epsilon_{Y}}\left(Y_{t}\right)=1\right)=P\left(D_{\epsilon_{X}}\left(X_{t}\right)=1\right)\cdot P\left(D_{\epsilon_{Y}}\left(Y_{t}\right)=1\right).$$

If $\epsilon_{X}=\epsilon_{Y}=0$ and $X_{t}=W_{t}$, $X_{t-1}=W_{t-1}$, $Y_{t}=T_{t}$, $Y_{t-1}=T_{t-1}$, the following paradox occurs

(11) $$1\overset{1}{\approx }P^{*}\left(D_{0}\left(W_{t}\right)=1|D_{0}\left(T_{t}\right)=1\right)\overset{2}{\approx }P^{*}\left(D_{0}\left(W_{t}\right)=1\right).$$

Equation (11) is a paradox in the following sense. If $\epsilon_{W}=\epsilon_{T}=0$, both $W_{t}$ and $T_{t}$ change in most trials between times t−1 and *t*, which makes the left side of Equation (11) true. In other words,

$$1\overset{1}{\approx }P^{*}\left(D_{0}\left(W_{t}\right)=1|D_{0}\left(T_{t}\right)=1\right).$$

However, if no restrictions are placed on weight and traffic measurements through $\epsilon_{W}$ and $\epsilon_{T}$, $W_{t}$ does not depend on $T_{t}$, which makes the right side of Equation (11) true. In other words,

(12) $$P^{*}\left(D_{0}\left(W_{t}\right)=1|D_{0}\left(T_{t}\right)=1\right)\overset{2}{\approx }P^{*}\left(D_{0}\left(W_{t}\right)=1\right).$$

Thus, the thresholds for weight and traffic, $\epsilon_{W}$ and $\epsilon_{T}$, must be above zero. Furthermore, the traffic threshold $\epsilon_{T}$ must be further constrained as

(13) $$\forall \epsilon_{T}>0:P\left(D_{\epsilon_{T}}\left(T_{t}\right)=1\right)>0,$$

because otherwise the joint probability of $\left\{D_{\epsilon_{W}}\left(W_{t}\right)=1\right\}$ and $\left\{D_{\epsilon_{T}}\left(T_{t}\right)=1\right\}$ is 0 whenever $P(D_{\epsilon_{T}}\left(T_{t}\right)=1)$ is 0. To establish the feasible values of $\epsilon_{W}$ and $\epsilon_{T}$, we used our datasets to compute the sets

(14) $$\begin{array}{ccc} \Delta_{T} & = & \{\Delta_{k}T_{t}:\forall t=1,2,...,\left\lfloor n/k\right\rfloor \};\\ \Delta_{W} & = & \{\Delta_{k}W_{t}:\forall t=1,2,...,\left\lfloor n/k\right\rfloor \}, \end{array}$$

where *n* is the total number of records in the dataset (see Table 1) and k∈{4,8,12,16,20,24}. Thus, since all the lags are 15 k, we tested the lags from 15×4 min (1 h) up to 15×24 min (6 h). For each hive, we computed the suprema (i.e., the upper bounds) as

(15) $$\begin{array}{ccc} \epsilon_{W}^{*} & = & \max_{\epsilon_{W}\in \Delta_{W}}\epsilon_{W};\\ \epsilon_{T}^{*} & = & \max_{\epsilon_{T}\in \Delta_{T}}\epsilon_{T} \end{array}$$

for each lag (1 h, 2 h, 3 h, 4 h, 5 h, 6 h), and 15 traffic measurements: 5 types of traffic (i.e., IN, OUT, TOT, IN-OUT, IN+OUT), 5 variances (i.e., $\sigma^{2}$(IN), $\sigma^{2}$(OUT), $\sigma^{2}$(TOT), $\sigma^{2}$(IN-OUT), $\sigma^{2}$(IN+OUT)), and 5 means (i.e., μ(IN), μ(OUT), μ(TOT), μ(IN-OUT), μ(IN+OUT)). We also tested the necessary condition for Independence (NCI) in Equation (10) on our dataset for each tuple of the three feasible values (i.e., *k*, $0<\epsilon_{W}<\epsilon_{W}^{*}$, $0<\epsilon_{T}<\epsilon_{T}^{*}$) by computing for each hive the threshold θ such that

(16) $$\max_{\theta }\left\{\right|P^{*}\left(D_{\epsilon_{W}}\left(W_{t}\right)=1,D_{\epsilon_{T}}\left(T_{t}\right)=1\right)-P^{*}\left(D_{\epsilon_{W}}\left(W_{t}\right)=1\right)P^{*}\left(D_{\epsilon_{T}}\left(T_{t}\right)=1\right)\left|\leq \theta \right\}.$$

in order to discover the upper bound of the difference between the joint and marginal probabilities. The value of θ in Expression (16), to which we refer as maxD, was computed for all 15 traffic measurements. We also computed the argmaxima of absolute differences between the joint probability (the left-hand side of Equation (10)) and the product of the marginal probabilities (the right-hand side of Equation (10)) for all hives.

### 2.6. $X^{2}$ Tests

The NCI implies a sufficiency criterion of the independence of $X_{t}$, $X_{t-1}$, $Y_{t}$, $Y_{t-1}$, which can be formulated as follows.

**A Sufficiency Condition for Independence (SCI):** Let $X_{t}$, $X_{t-1}$, $Y_{t}$, $Y_{t-1}$ are discrete independent random variables. If, for any $\epsilon_{X}$ and $\epsilon_{Y}$,

$$P\left(D_{\epsilon_{X}}\left(X_{t}\right)=1,D_{\epsilon_{Y}}\left(Y_{t}\right)=1\right)=P\left(D_{\epsilon_{X}}\left(X_{t}\right)=1\right)\cdot P\left(D_{\epsilon_{Y}}\left(Y_{t}\right)=1\right),$$

then $X_{t}$, $X_{t-1}$, $Y_{t}$, $Y_{t-1}$ are independent.

Since we cannot prove this criterion as a theorem, because, as of now, it is unclear to us how to formulate general, entomological realistic assumptions on the distributions of the variables, we executed the $\chi^{2}$ tests to estimate the independence of $W_{t}$, $W_{t-1}$, $T_{t}$, $T_{t-1}$ on our dataset. For each sample $\left(W_{1},T_{1}\right),\ldots ,\left(W_{n},T_{n}\right)$, we introduced *k* grouping intervals $\Delta_{1},\ldots ,\Delta_{k}$ for values $\Delta_{k}W$ and *m* grouping intervals $\nabla_{1},\ldots ,\nabla_{m}$ for $\Delta_{k}T$ values. We used the following hypotheses in the $\chi^{2}$ tests:

(17) $$\begin{array}{ccc} H_{\chi^{2},0}:p_{rc} & = & p_{r}\cdot p_{c};\\ H_{\chi^{2},1}:p_{rc} & \neq & p_{r}\cdot p_{c}, \end{array}$$

where $p_{rc}$ is the probability of an observation belonging to $\Delta_{r}\times \nabla_{c}$, $p_{r}$ is the probability of $\Delta_{k}W\in \Delta_{r}$, and $p_{c}$ is the probability of $\Delta_{k}T\in \nabla_{c}$. We split the domain of $\Delta_{k}W$ and $\Delta_{k}T$ into sub-intervals with the same probability to ensure that the probability of $\Delta_{k}W_{t}$ falling into any sub-interval is equal for all intervals. Since the true distributions of *W* and *T* are unknown, we separated both domains into the intervals with the the same number of counts from our dataset, which is the standard approach in $\chi^{2}$ tests [22]. The literature on the $\chi^{2}$ tests has two recommendations, which we used for all lags in our $\chi^{2}$ tests. The first recommendation, $REC_{1}$, is that each cell in the $\chi^{2}$ cumulative table for $\left(W_{t},T_{t}\right)$ contain at least 5 observations on average, which is a standard requirement for the practicability of $\chi^{2}$ tests [22]. The second recommendation, $REC_{2}$, is to split the domain of the investigated samples into a number of intervals *m* in accordance with the number of samples *n*. For example, if *n* is in [40,100), then m∈[7,9], if *n* is in [100,500), then m∈[8,12] [23]. If $REC_{2}$ is followed, the deviation of the histogram from the actual distribution density is minimal [24]. To estimate the impact of lags on the relationship between hive weight and traffic, we computed the $H_{\chi^{2},0}$ rejection ratios for all hives and lags from 1 h up to 6 h in 1 h increments. We also computed the correlation coefficients and their *p*-values for all hives and the same lags between $W_{t}$ and $T_{t}$ to see if these two different statistical methods agree on the impact.

## 3. Results

**Figure 1:**
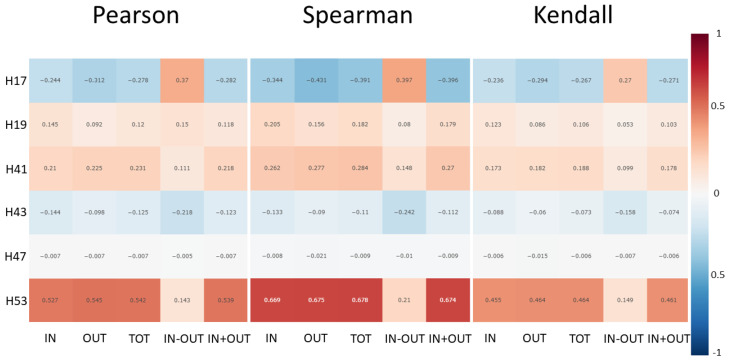
Correlation heat map of weight (W) and 5 types of traffic (T) on the x-axis for hives H17, H19, H41, H43, H47, and H53 on the y-axis; IN—incoming traffic; OUT—outgoing traffic; TOT—total traffic; IN-OUT—difference of IN and OUT; IN+OUT—sum of IN and OUT; TOT = IN + OUT + LAT (sum of IN, OUT, and LAT), where LAT—lateral traffic; IN, OUT, LAT, and TOT are non-negative integers.

Figure 1 shows the correlation heat map of Pearson, Spearman, Kendall for all monitored hives and five traffic types. Each cell of the heat map gives the computed coefficient value between IN, OUT, IN-OUT, IN+OUT, and TOT, on the one hand, and the weight of a given hive, on the other hand. Thus, in the bottom left cell (IN, H53), 0.527 is the Pearson coefficient between IN and *W* of hive H53. Table 2 gives the *p*-values for Pearson, Spearman, and Kendall for the monitored hives. Table 3 provides the suprema $\epsilon_{W}^{*}$ and $\epsilon_{T}^{*}$ for hive H17 computed by Equation (15). The suprema values for the other hives are given in the supplementary materials (see Tables S1–S5). For space considerations, we chose to present the results in terms of hive H17 as a representative of the group that included hives H17, H19, H41, H43, and H53, because these hives exhibited similar trends and patterns different from those of hive H47.

**Figure 2:**
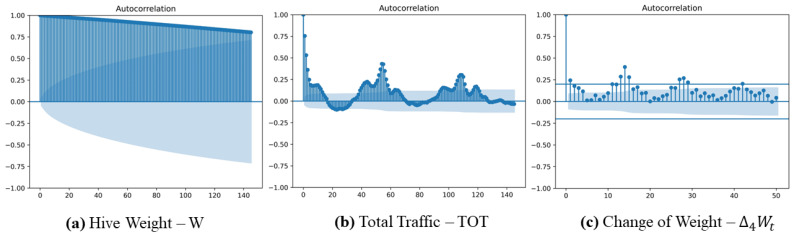
Autocorrelation plots of weight (**a**), total traffic (TOT) (**b**), and change in weight over 1 h (**c**) for hive H17 given using a standard python method *statsmodels.graphics.tsaplots*; the lags for (**a**,**b**) change from 0 to 145, where value 145 approximately equals 2.5 periods as 54 is the full number of records per day; the lag for (**c**) runs from 0 to 50 (≈3.5 periods) with a period of 12 (i.e., the number of data points per day for k=4); semi-transparent solid blue regions represent confidence intervals for ACF values.

The autocorrelation plots for weight and total traffic (TOT) for hive H17 are given in Figure 2. The plots for the other hives are in the supplementary materials (Figures S3–S7).

The ACF plots indicate that both weight and traffic include trend components insomuch as the amplitudes gradually decrease as the lag increases. Figure 2a,c show that trends and cycles are present in the weight data. Figures S3–S7 in the supplementary materials indicate that the weight and traffic of the other hives also exhibit trends and cyclical patterns. Figure 2b reflects a cyclical pattern in TOT with a period of 54 points (i.e., the full number of records per day) with the ACF peaks corresponding to L≈54 and L≈108.

The computation of Equation (16) on our dataset showed that if the difference between *t* and t−1 (i.e., the lag) was no longer than 6 h, then for any $0<\epsilon_{W}<\epsilon_{W}^{*}$ and $0<\epsilon_{T}<\epsilon_{T}^{*}$, the difference between the joint probability $P^{*}\left(D_{\epsilon_{W}}\left(W_{t}\right)=1,D_{\epsilon_{T}}\left(T_{t}\right)=1\right)$ and the marginal probabilities $P^{*}\left(D_{\epsilon_{W}}\left(W_{t}\right)=1\right)\cdot P^{*}\left(D_{\epsilon_{T}}\left(T_{t}\right)=1\right)$ did not exceed 0.15. In other words,

(18) $$|P^{*}\left(D_{\epsilon_{W}}\left(W_{t}\right)=1,D_{\epsilon_{T}}\left(T_{t}\right)=1\right)-P^{*}\left(D_{\epsilon_{W}}\left(W_{t}\right)=1\right)\cdot P^{*}\left(D_{\epsilon_{T}}\left(T_{t}\right)=1\right)|\leq 0.15.$$

**Figure 3:**
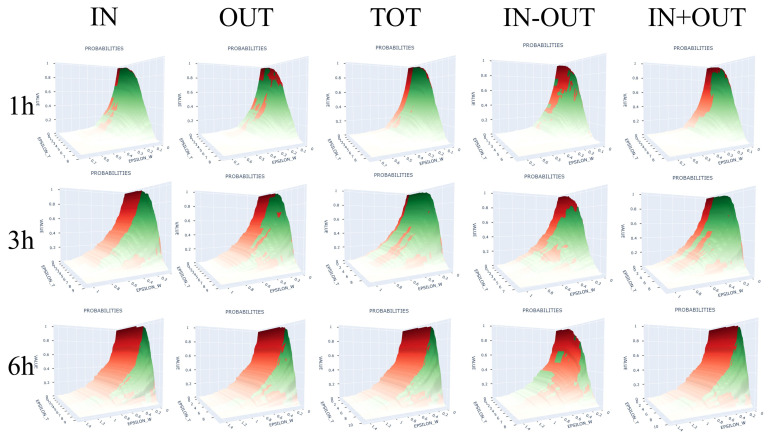
Joint probability values (green) and product of marginal probabilities (red) for incoming (IN), outgoing (OUT), total (TOT), difference of incoming and outgoing (IN-OUT), sum of incoming and outgoing (IN+OUT), and all values $0<\epsilon_{W}<\epsilon_{W}^{*}$ and $0<\epsilon_{T}<\epsilon_{T}^{*}$; IN, OUT, and TOT are non-negative integers.

Figure 3 shows the graphs of the joint and marginal probabilities for lags of 1 h, 3 h, and 6 h and five traffic types for all values of $0<\epsilon_{W}<\epsilon_{W}^{*}$ and $0<\epsilon_{T}<\epsilon_{T}^{*}$. Table 4, Tables S6 and S7 give the argmaxima of the absolute differences between the exact counts, variances, and means of incoming, outgoing, total, and lateral traffic, as well as the difference between incoming and outgoing traffic, the sum of incoming and outgoing traffic, and the corresponding value of $\epsilon_{W}$ and $\epsilon_{T}$ for hive H17.

Table 5, Tables S41 and S42 give the $\chi^{2}$ statistics *C* for the exact traffic counts, their variances, means, and different lags, and their *p*-values for hive H17.

Table 6 gives the traffic types and lags for which $H_{\chi^{2},0}$ was rejected at p≤0.05, which occurred in 142 out of 540 tested cases.

Table 7 presents the Pearson coefficients and the corresponding *p*-values for hive H17 for different lags. The analogous tables for the other hives are available in the supplementary materials (see Tables S58–S62).

Table 8, Table 9, Table 10, Table 11 and Table 12 show our counts of the frames of bees, mite drops, brood quality assessments, adult bee mass measurements, and the queen status inspections.

## 4. Discussion

**Figure 4:**
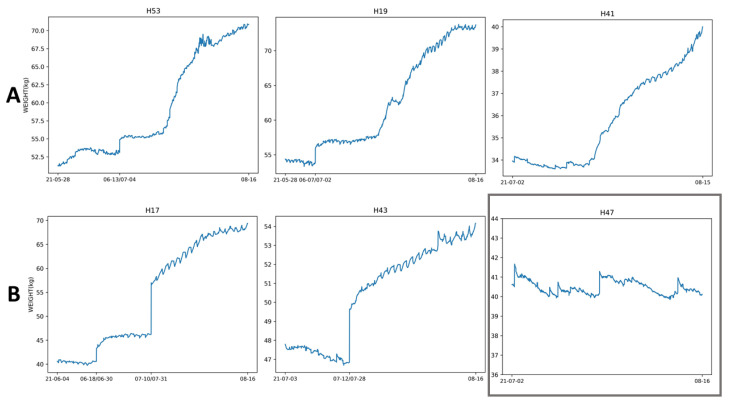
Weight (kg) vs. time of hives H17, H19, H41, H43, H47, and H53; first (leftmost) and last (rightmost) x-labels depict first and last day of time-aligned weights and video records; additional marks on x-axis in between leftmost and rightmost labels denote periods when videos were not taken due to hardware failures (e.g., 13 June/4 July on x-axis of H53 plot means that in H53 videos were not taken from 13 June 2021 up to 4 July 2021); row (**A**): weight vs. time for hives with positive correlation coefficients; row (**B**): weight vs. time with negative correlation coefficients; in row (**B**) weight vs. time plot of H47 is in gray box; H47 is the only hive for which $H_{\rho ,0}$ and $H_{\chi^{2},0}$ were not rejected.

Figure 1 shows that the correlation coefficients between weight and all traffic measurements, except for IN-OUT, are relatively close to each other. Hive H17 was the only hive with notable correlations between IN-OUT and weight for all three coefficients. Hive H53 showed strong correlations between weight and the four traffic measurements (IN, OUT, TOT, and IN+OUT). Hives H19 and H41 showed moderately positive correlations which were not as strong as those of hive H53. Hives H17 and H43 showed negative correlations, with H17 correlations being more negative than those of H43. H17 was the only hive for which the sign of the correlation coefficients between $W_{t}$ and IN-OUT were different from those of IN, OUT, TOT, and IN+OUT. All correlations of H47 were ≈0, which suggests that measured traffic and weight for this hive were uncorrelated. Table 2 shows that H47 was the only hive for which $H_{\rho ,0}$ was not rejected for any coefficient and any traffic type at p≤0.05. For all other hives, $H_{\rho ,0}$ was rejected at p≤0.05. H47 was the only hive whose weight did not increase with time. The weight of H47 oscillated between 40 and 41.5 kg throughout the experiment (see Figure 4). This observation leads to a conjecture that weight and traffic may not be correlated in hives that are not gaining weight, which merits further investigation. There may exist a traffic threshold per given lag below which a hive does not gain weight. Marceau et al. [6] experimentally concluded that for a positive daily gain the traffic must be at least 14,000 bees/h, but did not elaborate on whether this was mean traffic or real traffic. Our assumption of the independence and identical distribution of the values of $\Delta_{k}W_{t}$ (and $\Delta_{k}T_{t}$) on different days is reasonable insomuch as it is hard to assume that the differences in weight (and traffic) taken on a specific time interval on one day are related to the differences in weight (and traffic) taken on the same time interval but on a different day. More field data are required to investigate the independence and identical distribution of $\Delta_{k}W_{t}$ and $\Delta_{k}T_{t}$ for different lags.

Table 3, Table 4, Tables S6 and S7 for hive H17, the analogous tables for the other hives in the supplementary materials, and the plots in Figure 3 give an experimental validation of the result in Inequality (18) in that the difference between the joint and marginal probabilities was relatively small. In other words, traffic $W_{t}$ and weight $T_{t}$ might have been necessarily (but not sufficiently!) independent in the monitored hives so long as the lag did not exceed 6 h. The joint probabilities and the marginal probability products in Table 4 and Tables S6–S22 diverge by no more than 15% across all 15 traffic measurements for all hives (See Inequality (18)). The maximum absolute difference occurred mostly on the median values for weight and traffic. The divergence as a function of $\epsilon_{W}$ and $\epsilon_{T}$ reached its maximum when $\epsilon_{W}$ and $\epsilon_{T}$ were such that $P^{*}\left(D_{\epsilon_{W}}\left(W_{t}\right)=1\right)\approx P^{*}\left(D_{\epsilon_{T}}\left(T_{t}\right)=1\right)\approx 0.5$ (see Tables S23–S40). As the tables with $P^{*}$ at the extrema in the supplementary materials show, this observation held for all hives. Inequality (18) suggests that the events $\left\{\Delta_{k}W_{t}\geq \epsilon_{W}\right\}$ and $\left\{\Delta_{k}T_{t}\geq \epsilon_{T}\right\}$ are independent for any $\epsilon_{W}$ and $\epsilon_{T}$, for all 15 traffic measurements and all lags from 1 h up to 6 h. Consequently, the necessary condition of independence (NCI) of weight and traffic was experimentally verified on our dataset. Of course, this verification does not imply that weight and traffic *were* independent on the tested lags, because our $\chi^{2}$ results failed to verify the implied sufficiency condition for independence (SCI).

The results in Table 5 and Tables S41–S57 in the supplementary materials, and Table 6 indicate that there was an association between $W_{t}$ and traffic measurements at p≤0.05 for all hives. However, these results should be interpreted with caution because the $\chi^{2}$ tests fail to distinguish $H_{\chi^{2},0}$ from $H_{\chi^{2},1}$ if the probabilities of the observations falling into the partitioning intervals are the same for both hypotheses. On our dataset, the longer the lag was, the smaller the number of records for that lag were recorded. More field deployments are needed to increase the number of records for longer lags. Several rejections of $H_{\chi^{2},0}$ at smaller lags suggest that *W* and *T* may be correlated even on smaller lags. The hives whose weight and several traffic measures were related at lags of 1 h and 2 h (hives H17, H19, and H53) saw significant weight gains (+20 kg) for the entire monitored period of May–August 2021. This might indicate that the more frequently foragers leave the hive, the more weight the hive gains. It is unclear, however, whether the weight gain of a hive depends on the forager fly rate or on the efficiency of individual foragers (i.e., how much payload each forager brings back to the hive). Table 6 indicates that there is a relationship between traffic as measured by IN, TOT, IN+OUT, μ(OUT), μ(TOT), and μ(IN+OUT) and the weight for all hives.

The Pearson coefficients in Table 7 indicate that as the lag increased from 1 h to 6 h, the absolute value of Pearson increased, which means that on longer lags $W_{t}$ and $T_{t}$ became more correlated for hive H17 and the other hives (see the tables in the supplementary materials), except for H47. This observation, however, should not be interpreted as *causality*, because there may have been an underlying hidden variable (e.g., the declining health of the queen) that caused the two random variables to change in tandem. The *p*-values in Table 7 show that for hive H17, $H_{\rho ,0}$ was rejected for all lags. Similar observations (i.e., stronger Pearson correlation between $W_{t}$ and $T_{t}$ on longer lags, and $H_{\rho ,0}$ rejection for all lags) can be made on the analogous tables for all other hives, except for hive H47, in the supplementary materials (see Tables S58–S62). For H47, $H_{\rho ,0}$ was not rejected for any lags. H41 and H47 were the only two hives that did not have significant gains in both weight (see Figure 4) and bee mass (see Table 11) at the end of the experiment. The weight of H41 increased only by 6 kg, and the weight of H47 essentially remained the same. However, H41 had positive within-day correlations between weight and traffic, while the same correlations in H47 were negative and close to zero. H47 had lower counts of frames of bees, with the counts falling from 6 on 27 May 2021 to 5 on 13 August 2021 (see Table 8). H41 was the only other hive where the frames of bees fell from six to four during the same period. For H47, the mite drop started at 1.7 (the lowest of all hives) at the beginning of the monitored period and rose to 3.7 in August, 2021 (see Table 9). Table 10 shows that H47 and H41 were infected with the PMS and had spotty brood patterns. Table 11 shows that H47 had the lowest bee mass in June, 2021, and the second lowest bee mass at the end of the experiment in August, 2021, which may explain why H47 had the lowest mite drop. In H41, a supersedure queen was present in the hive for about one week. This queen was removed on 8 June 2021 (see Table 12) and replaced by a new one year old Italian queen from the same breeder from the queen bank at the USDA ARS Center in Tucson, AZ. The new queen might have been more productive than the removed queen, which may explain why H41 had positive and H47 negative correlations between weight and traffic in Figure 1. The supersedure queen may have mated with Africanized feral drones in Tucson, AZ, which may have affected traffic patterns in H41. In H47, an Italian queen from breeder 1 was removed on 19 May 2021 (four days after the start of the experiment) due to lack of productivity and replaced with a one-year-old Italian queen from breeder 2 from the same queen bank. Thus, H47 was the only hive where a one-year-old Italian queen from one breeder was replaced by a one-year-old Italian queen from a different breeder, which may have been a contributing factor in the lack of correlation between hive weight and traffic in this hive. All other queens survived and no other hives were re-queened during the monitored period.

Table 13 and Table 14 summarize the $H_{\chi^{2},0}$ rejection ratios for all lags. Table 13 takes into account the $\chi^{2}$ results for all lags, while Table 14 gives the results for the lags satisfying both recommendations, $REC_{1}$ and $REC_{2}$, for $\chi^{2}$ tests at the end of Section 2.6. Table 13 shows that as the lag increased from 1 h to 6 h, the frequency of $H_{\chi^{2},0}$ rejection also increased, indicating that for all hives $W_{t}$ and $T_{t}$ became more frequently associated as the lags became longer, which corroborates the result achieved with the Pearson coefficients in Table 7. The same tendency is observed in Table 14. Therefore, the observation that the strength of the correlation between $W_{t}$ and $T_{t}$ increased with the lag was confirmed by the correlation coefficients and $\chi^{2}$ results. Additional field deployments will enable us to collect more data in order to investigate which lags maximize weight–traffic correlation and to find computational methods for determining the optimal duration of traffic observations for accurate weight prediction from traffic. Table 15 gives the $H_{\chi^{2},0}$ rejection ratios for exact traffic measurements, traffic measurement variances, and traffic measurement means and indicates that the rejection ratios are basically the same for the three statistics, which suggests that traffic variance and traffic mean, when taken separately, do not affect the correlation between $W_{t}$ and $T_{t}$ more significantly than exact traffic counts. Thus, the latter may suffice in computational models that relate weight and traffic.

While the necessary condition for the independence (NCI) of weight and traffic was experimentally verified, the $\chi^{2}$ tests failed to verify the implied sufficiency condition for independence (SCI) for any lag. A possible explanation is that $D_{\epsilon }\left(\cdot \right)$ in Equation (8) divides the values of $\Delta_{k}W$ and $\Delta_{k}T$ into two categories, while the $\chi^{2}$ tests divide $\Delta_{k}Z_{t}$ into multiple (more than two) categories. The computed correlation coefficients and the executed $\chi^{2}$ tests indicate that the relation between total traffic (TOT) and weight $W_{t}$ and the sum of the incoming and outgoing traffic (IN+OUT) and $W_{t}$ are basically the same, which suggests that lateral traffic did not have a significant impact on weight change and may be omitted in computational models that relate weight and traffic. Additional field deployments will allow us curate larger datasets to test the strength of the relation and, as opportunity arises, discover its statistical or mathematical nature.

## 5. Conclusions

Hive weight $W_{t}$ and traffic $T_{t}$ were more correlated on longer lags. The strength of the correlation increased with the lag when estimated with Pearson, Spearman, and Kendall. The $\chi^{2}$ tests also showed that as the lag increased from 1 h to 6 h, the frequency of $H_{\chi^{2},0}$ rejection increased, indicating that $W_{t}$ and $T_{t}$ became more related on longer lags for all hives. This conclusion may not be causal, because there may have been underlying hidden variables (e.g., the health of the queen) that caused $W_{t}$ and $T_{t}$ to change in tandem. Our autocorrelation analysis suggests that both weight and total traffic exhibit trends and cyclical patterns. A feasible reason why some hives had positive and some hives had negative correlations between $W_{t}$ and $T_{t}$ may lie in the genetic differences between the queen lines from two U.S. breeders of Italian queens, which may warrant further investigation in the future. A number of factors could contribute to the lack of correlation between hive weight and traffic in H47: the replacement of the original queen with a queen from a different breeder, insignificant gain in bee mass, spotty brood patterns, and PMS infection. The spread in traffic and average traffic, when taken separately, did not affect the correlation of $W_{t}$ and $T_{t}$ more significantly than the exact traffic counts from videos. The correlation coefficients and the $\chi^{2}$ tests showed that the relation between total traffic (TOT) and weight $W_{t}$ and the sum of incoming and outgoing traffic (IN+OUT) and $W_{t}$ are basically the same. Thus, lateral traffic (LAT) did not have a significant impact on weight change and may be omitted in computational models that relate hive weight and traffic in the vicinity of the hive. We hope that our study and similar studies will eventually result in a methodology to separate the hive weight associated with traffic from the hive weight not associated with it, which, in turn, will lead to the construction of computational models that predict hive weight from hive traffic.

## Figures and Tables

**Table 1:** A quantitative summary of the curated dataset; the number of records *n* for each hive specifies the number of time-aligned weight and traffic measurements.

*Hive*	*Num Records (n)*	*Num Recorded Days*	*Mean Records per Day*
H17	1838	36	51
H19	3019	56	54
H41	1842	40	46
H43	1630	30	54
H47	2518	46	55
H53	2506	56	45
Total	13,353	56	≈50.83

**Table 2:** Pearson, Spearman, and Kendall *p*-values rounded to 3 decimal places for hives H17, H19, H41, H43, H47, and H53 and five traffic types: IN (incoming), OUT (outgoing), TOT (total), IN-OUT (difference of IN and OUT), and IN+OUT (sum of IN and OUT), where TOT = IN+OUT+LAT and LAT is lateral traffic; IN, OUT, LAT, and TOT are non-negative integers; *p*-values for H47 are bolded, because it was the only hive for which $H_{\rho ,0}$ was not rejected at p≤0.05 for any coefficient and any traffic type; HXX refers to H17, H19, H41, H43, and H53, because their *p*-values were identically 0.

*Hive*			*Pearson*					*Spearman*					*Kendall*		
	IN	OUT	TOT	IN-OUT	IN+OUT	IN	OUT	TOT	IN-OUT	IN+OUT	IN	OUT	TOT	IN-OUT	IN+OUT
H47	0.724	**0.715**	**0.721**	**0.801**	**0.719**	**0.675**	**0.284**	**0.661**	**0.629**	**0.656**	**0.661**	**0.265**	**0.651**	**0.604**	**0.639**
HXX	0	0	0	0	0	0	0	0	0	0	0	0	0	0	0

**Table 3:** Suprema $\epsilon_{W}^{*}$ and $\epsilon_{T}^{*}$ computed according to Equation (15), for hive H17 and for 5 types of traffic, 5 variances, and 5 means; IN—incoming traffic; OUT—outgoing traffic; TOT—total traffic; LAT—lateral traffic; IN—OUT (difference between IN and OUT), IN + OUT (sum of IN and OUT); TOT = IN + OUT + LAT; W column gives $\epsilon_{W}^{*}$ for weight measurements and corresponding lags; IN, OUT, TOT, and LAT are non-negative integers; exact measurement columns give $\epsilon_{W}^{*}$ for exact measurements of IN, OUT, TOT, IN-OUT, IN+OUT and lags; variance columns give $\epsilon_{T}^{*}$ for $\sigma^{2}$(X), where X ∈ {IN, OUT, TOT, IN-OUT, IN+OUT} and lags; mean columns give $\epsilon_{T}^{*}$ for μ(X), where where X ∈ {IN, OUT, TOT, IN-OUT, IN+OUT}.

Lag			Exact	Measurement					Variance					Mean		
(hours)	W	IN	OUT	TOT	IN-OUT	IN+OUT	IN	OUT	TOT	IN-OUT	IN+OUT	IN	OUT	TOT	IN-OUT	IN+OUT
1	0.754	7.856	7.968	8.707	6.585	8.606	11.85	11.949	13.459	10.899	13.261	6.581	6.47	7.321	5.198	7.22
2	0.807	8.463	8.659	9.328	7.223	9.246	12.083	12.051	13.652	10.4	13.45	6.58	6.383	7.249	5.143	7.167
3	1.056	8.783	9.013	9.68	7.494	9.597	11.873	11.865	13.445	10.051	13.251	6.528	6.298	7.195	5.01	7.113
4	1.344	8.988	9.179	9.865	7.602	9.781	11.814	11.792	13.37	9.776	13.185	6.406	6.215	7.093	4.83	7.009
5	1.43	9.129	9.326	10.008	7.719	9.926	11.762	11.753	13.324	9.608	13.14	6.331	6.133	7.012	4.723	6.93
6	1.455	9.254	9.446	10.129	7.795	10.048	11.672	11.673	13.238	9.455	13.055	6.268	6.076	6.951	4.617	6.87

**Table 4:** The maxima and argmaxima of the absolute difference between the joint probability and the product of marginal probabilities of exact traffic counts and different lags for hive H17; each entry has format (maxD, $\epsilon_{W}$, $\epsilon_{T}$); IN—incoming traffic, OUT—outgoing traffic; TOT—total traffic; IN-OUT—difference between IN and OUT; IN+OUT—sum of IN+OUT; IN, OUT, TOT, IN-OUT, and IN+OUT are integers.

Lag			*maxD*, $\epsilon_{W}$, $\epsilon_{T}$		
(hours)	IN	OUT	TOT	IN-OUT	IN+OUT
1	(0.072, 0.051, 5.416)	(0.059, 0.059, 6.227)	(0.068, 0.051, 6.339)	(0.029, 0.077, 3.871)	(0.066, 0.051, 6.382)
2	(0.059, 0.181, 7.050)	(0.051, 0.145, 7.098)	(0.060, 0.145, 7.848)	(0.038, 0.284, 4.727)	(0.054, 0.145, 7.813)
3	(0.051, 0.226, 6.701)	(0.042, 0.214, 6.347)	(0.046, 0.228, 7.496)	(0.044, 0.054, 4.754)	(0.044, 0.228, 7.419)
4	(0.071, 0.602, 7.467)	(0.065, 0.602, 7.505)	(0.069, 0.602, 8.305)	(0.045, 0.049, 5.182)	(0.069, 0.602, 8.234)
5	(0.055, 0.080, 8.027)	(0.060, 0.080, 8.019)	(0.058, 0.314, 8.971)	(0.078, 0.574, 4.963)	(0.060, 0.295, 8.895)
6	(0.077, 0.181, 7.840)	(0.070, 0.113, 7.946)	(0.083, 0.181, 8.695)	(0.053, 0.493, 6.435)	(0.078, 0.181, 8.627)

**Table 5:** Chi-square statistics and *p*-values of exact traffic counts for different lags for hive H17; 3 *p* values smaller than 0.05 are bolded; IN—incoming traffic; OUT—outgoing traffic; TOT—total traffic.

Lag			*C* and *p*-Value		
(hours)	IN	OUT	TOT	IN-OUT	IN+OUT
1	(88.523, **0.023**)	(77.522, 0.119)	(90.778, **0.016**)	(51.015, 0.880)	(94.585, **0.008**)
2	(35.668, 0.077)	(27.817, 0.316)	(36.710, 0.061)	(30.741, 0.198)	(33.929, 0.109)
3	(16.438, 0.423)	(14.751, 0.543)	(16.985, 0.387)	(26.175, 0.052)	(15.488, 0.489)
4	(24.382, 0.081)	(22.313, 0.133)	(24.379, 0.082)	(15.655, 0.477)	(20.985, 0.179)
5	(7.203, 0.616)	(12.325, 0.196)	(7.551, 0.580)	(10.224, 0.333)	(7.551, 0.580)
6	(10.964, 0.278)	(7.732, 0.561)	(9.093, 0.429)	(13.680, 0.134)	(8.386, 0.496)

**Table 6:** Lags for which $H_{\chi^{2},0}$ was rejected for monitored hives; IN—incoming traffic; OUT—outgoing traffic; TOT—total traffic; $\sigma^{2}$(X)—variance of X; μ(X)—mean of X; ⋄ means that $H_{\chi^{2},0}$ was not rejected for any lag; bolded lags are the lags for which both recommendations at the end of Section 2.6 are satisfied.

Traffic Measurement	H17	H19	H41	H43	H47	H53
IN	**1**	5,6	5,6	5	4,5	4,5,6
OUT	⋄	5,6	5,6	5,6	5	4,5,6
TOT	**1**	5,6	5,6	5,6	5	4,5,6
IN-OUT	⋄	5,6	6	⋄	5	⋄
IN+OUT	**1**	5,6	5,6	5	5	4,5,6
$\sigma^{2}$(IN)	**1**,2,3,6	**3**,5	6	⋄	3,4,5,6	**2**,4,5,6
$\sigma^{2}$(OUT)	**1**,2,3,6	**2**	⋄	⋄	⋄	**2**,4,5,6
$\sigma^{2}$(TOT)	**1**,3	**1**	6	⋄	3,5,6	**2**,4,5,6
$\sigma^{2}$(IN-OUT)	3,4	4	3	⋄	⋄	5,6
$\sigma^{2}$(IN+OUT)	**1**,2,3	⋄	6	⋄	3,6	4,5,6
μ(IN)	⋄	6	6	⋄	5,6	4,5,6
μ(OUT)	**1**	6	5,6	6	4,5	4,5,6
μ(TOT)	**1**,2	6	4,6	**1**,6	5,6	4,5,6
μ(IN-OUT)	⋄	**3**,6	⋄	⋄	4,5,6	4
μ(IN+OUT)	**1**,2	6	6	6	5,6	**1**,4,5,6

**Table 7:** Pearson coefficients and corresponding *p*-values of different types of traffic measurements and different lags for hive H17; each cell is a tuple (c,p) where *c* is Pearson and *p* is *p*-value; IN—incoming traffic; OUT—outgoing; TOT—total.

Lag (in hours)	IN	OUT	TOT	IN-OUT	IN+OUT
1	(−0.288, 1.33 × 10${}^{-9}$)	(−0.362, 1.17 × 10${}^{-14}$)	(−0.324, 6.76 × 10${}^{-12}$)	(0.490, 3.92 × 10${}^{-27}$)	(−0.329, 3.06 × 10${}^{-12}$)
2	(−0.328, 1.98 × 10${}^{-6}$)	(−0.406, 2.15 × 10${}^{-9}$)	(−0.367, 8.61 × 10${}^{-8}$)	(0.539, 1.53 × 10${}^{-16}$)	(−0.372, 5.34 × 10${}^{-8}$)
3	(−0.367, 1.64 × 10${}^{-5}$)	(−0.445, 1.01 × 10${}^{-7}$)	(−0.406, 1.50 × 10${}^{-6}$)	(0.579, 4.22 × 10${}^{-13}$)	(−0.411, 1.09 × 10${}^{-6}$)
4	(−0.394, 6.03 × 10${}^{-5}$)	(−0.480, 5.82 × 10${}^{-7}$)	(−0.436, 7.10 × 10${}^{-6}$)	(0.627, 4.71 × 10${}^{-12}$)	(−0.442, 5.11 × 10${}^{-6}$)
5	(−0.399, 8.17 × 10${}^{-4}$)	(−0.483, 3.55 × 10${}^{-5}$)	(−0.440, 1.95 × 10${}^{-4}$)	(0.628, 1.24 × 10${}^{-8}$)	(−0.446, 1.54 × 10${}^{-4}$)
6	(−0.496, 4.17 × 10${}^{-5}$)	(−0.582, 7.16 × 10${}^{-7}$)	(−0.539, 6.07 × 10${}^{-6}$)	(0.716, 6.08 × 10${}^{-11}$)	(−0.545, 4.71 × 10${}^{-6}$)

**Table 8:** Frames of bees in monitored hives; frames of bees is a visual count of frames completely covered with bees on both sides; all inspection dates in columns were in 2021; NA—not available.

*Hive*	*27 May*	*8 June*	*30 July*	*28 July*	*13 August*
H17	8	9	13	18	18
H19	12	11	13	14	18
H41	6	6	4	4	4
H43	7	NA	7	8	15
H47	6	NA	4	3	5
H53	10	8	7	10	15

**Table 9:** Mite drop measurements in monitored hives; mite drop is the mean number of mites per day on a sticky board under the hive; all periods in columns refer to 2021; the start and end of each period are in the month/day format; a new period started on 7 August 2021 after new Apivar strips were installed in monitored hives; the last row represents Apivar treatment periods; Apivar is a polymer strip used to treat Varroa mites.

*Hive*	*27 May–7 June*	*7 June–6 July*	*6 July–8 July*	*8 July–16 August*
H17	2.3	7	9.5	19.3
H19	13	26	42	29.7
H41	3.7	2.5	7.5	4.7
H43	6.3	6	11	8.3
H47	1.7	6.5	4.5	3.7
H53	10	9	17	10.3
	*Pre-treatment*	*Mite treatment*		*Post-treatment*

**Table 10:** Brood quality measurements in monitored hives; STR—straight; SPT—spotty; PMS—parasitic mite syndrome; NA—not available; colonies with PMS have white larvae that appear chewed or sunken on the side of the cell.

*Hive*	*27 May*	*8 June*	*30 July*	*28 July*	*13 August*
H17	STR	STR	STR	STR	STR
H19	STR	STR	STR	STR	STR
H41	SPT	PMS	SPT	STR/SPT	SPT
H43	NA	NA	STR	STR/SPT	STR/SPT
H47	NA	NA	SPT/PMS	SPT/PMS	SPT
H53	STR	STR	SPT	STR	SPT

**Table 11:** Bee mass (kg) in monitored hives measured using the method in [9]; N/A—not applicable.

*Hive*	*8 June 2021*	*30 June 2021*	*13 August 2021*
H17	2.32	N/A	4.42
H19	N/A	2.12	4.10
H41	0.86	N/A	1.32
H43	N/A	2.05	3.15
H47	N/A	0.79	1.45
H53	N/A	1.36	3.08

**Table 12:** Queen status inspections of monitored hives; all dates in columns were in 2021; blu—queen from breeder 1 colored blue; gr—queen from breeder 2 colored green; yel—queen from breeder 2 colored yellow; Q+—queen spotted; Q+?—queen not spotted but its presence is clear from eggs in cells; Q?—queen not spotted; SSQR—supersedure queen removed; QR—queen spotted and removed; N/A – not available.

*Hive*	*19 May*	*27 May*	*8 June*	*30 June*	*28 July*	*13 August*
H17	Q+blu	Q+blu	Q+blu	Q+blu	Q+?	Q+blu
H19	Q+yel	Q+?	Q+yel	Q+yel	Q+yel	Q?
H41	Q+yel	Q+yel	SSQR	Q+yel	Q+yel	Q+yel
H43	Q+blu	Q?	N/A	Q+blu	Q+blu	Q?
H47	QR+blu	Q+yel	N/A	Q+yel	Q+yel	Q+yel
H53	Q+gr	Q+gr	Q+gr	Q+gr	Q+gr	Q+gr

**Table 13:** Lags and $H_{\chi^{2},0}$ rejection ratios for all monitored hives.

Lag (in Hours)	$H_{\chi^{2},0}$-Rejected/Number of Tests
1	13/90 ≈ 14.4%
2	9/90 = 10.0%
3	11/90 ≈ 12.2%
4	20/90 ≈ 22.2%
5	40/90 ≈ 44.4%
6	49/90 ≈ 54.4%

**Table 14:** Lags and $H_{\chi^{2},0}$ rejection ratios for which the domains of $W_{t}$ and $T_{t}$ were split into the numbers of intervals satisfying the recommendations $REC_{1}$ and $REC_{2}$.

Lag (in Hours)	$H_{\chi^{2},0}$-Rejected/Number of Tests
1	13/90 ≈ 14.4%
2	4/45 ≈ 8.9%
3	2/15 ≈ 13.3%

**Table 15:** $H_{\chi^{2},0}$ rejection ratios for exact traffic measurements (i.e., counts), traffic measurement variances, and traffic measurement means.

Traffic Measurements	$H_{\chi^{2},0}$-Rejected/Number of Tests
Exact traffic measurements	46/180 ≈ 25.6%
Traffic measurement variances ($\sigma^{2}$)	50/180 ≈ 27.8%
Traffic measurement means (μ)	46/180 ≈ 25.6%

## Data Availability

Details regarding where data supporting reported results can be found are given in the supplementary materials.

## Supplementary Materials

The following supplementary materials are available at https://www.mdpi.com/article/10.3390/s22134824/s1: (1) the PDF with three video sets that illustrate how the BeePIV algorithm processes videos with different levels of bee traffic; Tables S1–S62 with additional hive-specific correlation coefficients and p-values; and Figures S1–S7 that illustrate additional hive-specific aspects of autocorrelation of weight and traffic; (2) six datasets of weight and traffic which we used for the reported research.

## Abbreviations

The following abbreviations are used in this manuscript:

EBM	Electronic beehive monitoring
USDA-ARS	United States Department of Agriculture-Agricultural Research Service
TB	Terabyte = 1024 gigabytes
USB	Universal serial bus
kg, g	Kilogram, gram
km, m	Kilometer, meter
h	Hour
°C	Degree Celsius
MP4	MPEG-4 Part 14: Motion Picture Experts Group software for real-time data streams
ID	Identification
PIV	Particle image velocimetry
CSV	Comma-separated values
IN	Incoming bee traffic (bees flying toward the hive)
OUT	Outgoing bee traffic (bees flying away from the hive)
LAT	Lateral bee traffic (bees flying in parallel to the hive)
TOT	Total bee traffic in the vicinity of the hive
NCI	Necessary condition for independence
SCI	Sufficiency condition for independence
REC	Recommendation

## Appendix A

Suppose that Table A1 is our dataset. If we want to measure the change in the exact counts with the lag of one hour, then the exact counts are as follows.

$$\begin{array}{ccccc} \Delta_{4}W_{1} & = & |W_{1+1\cdot 4}-W_{1+(1-1)4}\left|=\right|W_{5}-W_{1}|=5-2=3; & \; & \\ \Delta_{4}{IN}_{1} & = & |{IN}_{1+(1-1)4}+{IN}_{2+(1-1)4}+{IN}_{3+(1-1)4}+{IN}_{1\cdot 4}-0|= & \; & \\ \; & = & |{IN}_{1}+{IN}_{2}+{IN}_{3}+{IN}_{4}|=20+12+10+18=60; & \; & \\ \Delta_{4}{OUT}_{1} & = & |{OUT}_{1}+{OUT}_{2}+{OUT}_{3}+{OUT}_{4}-0|=10+6+15+9=40; & \; & \\ \Delta_{4}{TOT}_{1} & = & |{TOT}_{1}+{TOT}_{2}+{TOT}_{3}+{TOT}_{4}-0|=35+19+29+30=113; & \; & \\ \Delta_{4}{IN-OUT}_{1} & = & |\left({IN}_{1}-{OUT}_{1}\right)+\left({IN}_{2}-{OUT}_{2}\right)+\left({IN}_{3}-{OUT}_{3}\right)+\left({IN}_{4}-{OUT}_{4}\right)-0|= & \; & \\ \; & = & (20-10)+(12-6)+(10-15)+(18-9)=10+6-5+9=20; & \; & \\ \Delta_{4}{IN+OUT}_{1} & = & |\left({IN}_{1}+{OUT}_{1}\right)+\left({IN}_{2}+{OUT}_{2}\right)+\left({IN}_{3}+{OUT}_{3}\right)+\left({IN}_{4}+{OUT}_{4}\right)-0| & \; & \\ \; & = & (20+10)+(12+6)+(10+15)+(18+9)=30+18+25+27=100; & \; & \\ \Delta_{4}W_{2} & = & |W_{1+2\cdot 4}-W_{1+(2-1)4}\left|=\right|W_{9}-W_{5}|=|3-5|=2; & \; & \\ \Delta_{4}{IN}_{2} & = & |{IN}_{1+(2-1)4}+{IN}_{2+(2-1)4}+{IN}_{3+(2-1)4}+{IN}_{2\cdot 4}-{IN}_{1+(1-1)4}-{IN}_{2+(1-1)4}- & \; & \\ \; & - & {IN}_{3+(1-1)4}-{IN}_{1\cdot 4}\left|=\right|{IN}_{5}+{IN}_{6}+{IN}_{7}+{IN}_{8}-{IN}_{1}-{IN}_{2}-{IN}_{3}-{IN}_{4}|= & \; & \\ \; & = & |13+8+11+17-20-12-10-18|=|49-60|=11; & \; & \\ \Delta_{4}{OUT}_{2} & = & |{OUT}_{5}+{OUT}_{6}+{OUT}_{7}+{OUT}_{8}-{OUT}_{1}-{OUT}_{2}-{OUT}_{3}-{OUT}_{4}|= & \; & \\ \; & = & |8+7+5+10-10-6-15-9|=|30-40|=10; & \; & \\ \Delta_{4}{TOT}_{2} & = & |{TOT}_{5}+{TOT}_{6}+{TOT}_{7}+{TOT}_{8}-{TOT}_{1}-{TOT}_{2}-{TOT}_{3}-{TOT}_{4}|= & \; & \\ \; & = & |26+17+18+34-35-19-29-30|=|95-113|=18; & \; & \\ \Delta_{4}{IN-OUT}_{2} & = & |\left({IN}_{5}-{OUT}_{5}\right)+\left({IN}_{6}-{OUT}_{6}\right)+\left({IN}_{7}-{OUT}_{7}\right)+\left({IN}_{8}-{OUT}_{8}\right)- & \; & \\ \; & - & \left({IN}_{1}-{OUT}_{1}\right)-\left({IN}_{2}-{OUT}_{2}\right)-\left({IN}_{3}-{OUT}_{3}\right)-\left({IN}_{4}-{OUT}_{4}\right)|= & \; & \\ \; & = & |(13-8)+(8-7)+(11-5)+(17-10)-(20-10)-(12-6)- & \; & \\ \; & - & (10-15)-(18-9)|=|5+1+6+7-10-6+5-9|=|-1|=1; & \; & \\ \Delta_{4}{IN+OUT}_{2} & = & |\left({IN}_{5}+{OUT}_{5}\right)+\left({IN}_{6}+{OUT}_{6}\right)+\left({IN}_{7}+{OUT}_{7}\right)+\left({IN}_{8}+{OUT}_{8}\right)- & \; & \\ \; & - & \left({IN}_{1}+{OUT}_{1}\right)-\left({IN}_{2}+{OUT}_{2}\right)-\left({IN}_{3}+{OUT}_{3}\right)-\left({IN}_{4}+{OUT}_{4}\right)|= & \; & \\ \; & = & |(13+8)+(8+7)+(11+5)+(17+10)-(20+10)-(12+6)- & \; & \\ \; & - & (10+15)-(18+9)|=|21+15+16+27-30-18-25-27|= & \; & \\ \; & = & |-21|=21. & \; & \end{array}$$

**Table A1:** Example dataset with nine records of W—weight, IN—incoming traffic, OUT—outgoing traffic, LAT—lateral traffic, and TOT—total traffic; TOT = IN + OUT + LAT.

TIME STEP	W	IN	OUT	LAT	TOT
1	2	20	10	5	35
2	3	12	6	1	19
3	1	10	15	4	29
4	4	18	9	3	30
5	5	13	8	5	26
6	2	8	7	2	17
7	1	11	5	2	18
8	1	17	10	7	34
9	3	3	14	9	26

If we want to measure the change in the variation of measurements IN,OUT,TOT,IN−OUT, and IN+OUT with the lag of one hour, then the variations are as follows.

$$\begin{array}{lll} \Delta_{4}\sigma^{2}{\left(IN\right)}_{1} & = & |\sigma^{2}\left({IN}_{1},{IN}_{2},{IN}_{3},{IN}_{4}\right)-0|=\sigma^{2}\left(20,12,10,18\right)=17;\;\\ \Delta_{4}\sigma^{2}{\left(OUT\right)}_{1} & = & \sigma^{2}\left({OUT}_{1},{OUT}_{2},{OUT}_{3},{OUT}_{4}\right)=\sigma^{2}\left(10,6,15,9\right)=10.5;\;\\ \Delta_{4}\sigma^{2}{\left(TOT\right)}_{1} & = & \sigma^{2}\left({TOT}_{1},{TOT}_{2},{TOT}_{3},{TOT}_{4}\right)=\sigma^{2}\left(35,19,29,30\right)=33.6875;\;\\ \Delta_{4}\sigma^{2}{\left(IN-OUT\right)}_{1} & = & \sigma^{2}\left({IN}_{1}-{OUT}_{1},{IN}_{2}-{OUT}_{2},{IN}_{3}-{OUT}_{3},{IN}_{4}-{OUT}_{4}\right)=\;\\ & = & \sigma^{2}\left(20-10,12-6,10-15,18-9\right)=\sigma^{2}\left(10,6,-5,9\right)=35.5;\;\\ \Delta_{4}\sigma^{2}{\left(IN+OUT\right)}_{1} & = & \sigma^{2}\left({IN}_{1}+{OUT}_{1}\right),{IN}_{2}+{OUT}_{2},{IN}_{3}+{OUT}_{3},{IN}_{4}+{OUT}_{4})=\;\\ & = & \sigma^{2}\left(30,18,25,27\right)=19.5;\;\\ \Delta_{4}\sigma^{2}{\left(IN\right)}_{2} & = & |\sigma^{2}\left({IN}_{5},{IN}_{6},{IN}_{7},{IN}_{8}\right)-\sigma^{2}\left({IN}_{1},{IN}_{2},{IN}_{3},{IN}_{4}\right)|=\\ & = & |\sigma^{2}\left(13,8,11,17\right)-\sigma^{2}\left(20,12,10,18\right)|=|10.6875-17|=6.3125;\;\\ \Delta_{4}\sigma^{2}{\left(OUT\right)}_{2} & = & |\sigma^{2}\left({OUT}_{5},{OUT}_{6},{OUT}_{7},{OUT}_{8}\right)-\sigma^{2}\left({OUT}_{1},{OUT}_{2},{OUT}_{3},{OUT}_{4}\right)|=\;\\ & = & |\sigma^{2}\left(8,7,5,10\right)-\sigma^{2}\left(10,6,15,9\right)|=7.25;\;\\ \Delta_{4}\sigma^{2}{\left(TOT\right)}_{2} & = & |\sigma^{2}\left({TOT}_{5},{TOT}_{6},{TOT}_{7},{TOT}_{8}\right)-\sigma^{2}\left({TOT}_{1},{TOT}_{2},{TOT}_{3},{TOT}_{4}\right)|=\;\\ & = & |\sigma^{2}\left(26,17,18,34\right)-\sigma^{2}\left(35,19,29,30\right)|=13.5;\;\\ \Delta_{4}\sigma^{2}{\left(IN-OUT\right)}_{2} & = & |\sigma^{2}\left({IN}_{5}-{OUT}_{5},{IN}_{6}-{OUT}_{6},{IN}_{7}-{OUT}_{7},{IN}_{8}-{OUT}_{8}\right)-\;\\ & - & \sigma^{2}\left({IN}_{1}-{OUT}_{1},{IN}_{2}-{OUT}_{2},{IN}_{3}-{OUT}_{3},{IN}_{4}-{OUT}_{4}\right)|=\;\\ & = & |\sigma^{2}\left(5,1,6,7\right)-\sigma^{2}\left(10,6,-5,9\right)|=30.3125;\;\\ \Delta_{4}\sigma^{2}{\left(IN+OUT\right)}_{2} & = & |\sigma^{2}\left({IN}_{5}+{OUT}_{5},{IN}_{6}+{OUT}_{6},{IN}_{7}+{OUT}_{7},{IN}_{8}+{OUT}_{8}\right)-\;\\ & - & \sigma^{2}\left({IN}_{1}+{OUT}_{1},{IN}_{2}+{OUT}_{2},{IN}_{3}+{OUT}_{3},{IN}_{4}+{OUT}_{4}\right)|=\;\\ & = & |\sigma^{2}\left(21,15,16,27\right)-\sigma^{2}\left(30,18,25,27\right)|=3.1875.\; \end{array}$$

The measure of the change in the mean of IN,OUT,TOT,IN−OUT, and IN+OUT with the lag of one hour are as follows.

$$\begin{array}{ccccc} \Delta_{4}\mu {\left(IN\right)}_{1} & = & |\mu \left({IN}_{1},{IN}_{2},{IN}_{3},{IN}_{4}\right)-0|=\mu \left(20,12,10,18\right)=15; & \; & \\ \Delta_{4}\mu {\left(OUT\right)}_{1} & = & \mu \left({OUT}_{1},{OUT}_{2},{OUT}_{3},{OUT}_{4}\right)=\mu \left(10,6,15,9\right)=10; & \; & \\ \Delta_{4}\mu {\left(TOT\right)}_{1} & = & \mu \left({TOT}_{1},{TOT}_{2},{TOT}_{3},{TOT}_{4}\right)=\mu \left(35,19,29,30\right)=28.25; & \; & \\ \Delta_{4}\mu {\left(IN-OUT\right)}_{1} & = & \mu ({IN}_{1}-{OUT}_{1},{IN}_{2}-{OUT}_{2},{IN}_{3}-{OUT}_{3},{IN}_{4}-{OUT}_{4})= & \; & \\ \; & = & \mu (20-10,12-6,10-15,18-9)=\mu (10,6,-5,9)=5; & \; & \\ \Delta_{4}\mu {\left(IN+OUT\right)}_{1} & = & \mu \left({IN}_{1}+{OUT}_{1}\right),{IN}_{2}+{OUT}_{2},{IN}_{3}+{OUT}_{3},{IN}_{4}+{OUT}_{4})= & \; & \\ \; & = & \mu (30,18,25,27)=25; & \; & \\ \Delta_{4}\mu {\left(IN\right)}_{2} & = & |\mu \left({IN}_{5},{IN}_{6},{IN}_{7},{IN}_{8}\right)-\mu \left({IN}_{1},{IN}_{2},{IN}_{3},{IN}_{4}\right)|= & \; & \\ \; & = & |\mu (13,8,11,17)-\mu (20,12,10,18)|=|12.25-15|=2.75; & \; & \\ \Delta_{4}\mu {\left(OUT\right)}_{2} & = & |\mu \left({OUT}_{5},{OUT}_{6},{OUT}_{7},{OUT}_{8}\right)-\mu \left({OUT}_{1},{OUT}_{2},{OUT}_{3},{OUT}_{4}\right)|= & \; & \\ \; & = & |\mu (8,7,5,10)-\mu (10,6,15,9)|=|7.5-10|=2.5; & \; & \\ \Delta_{4}\mu {\left(TOT\right)}_{2} & = & |\mu \left({TOT}_{5},{TOT}_{6},{TOT}_{7},{TOT}_{8}\right)-\mu \left({TOT}_{1},{TOT}_{2},{TOT}_{3},{TOT}_{4}\right)|= & \; & \\ \; & = & |\mu (26,17,18,34)-\mu (35,19,29,30)|=|23.75-28.25|= & \; & \\ \; & = & 4.5; & \; & \\ \Delta_{4}\mu {\left(IN-OUT\right)}_{2} & = & |\mu ({IN}_{5}-{OUT}_{5},{IN}_{6}-{OUT}_{6},{IN}_{7}-{OUT}_{7},{IN}_{8}-{OUT}_{8})- & \; & \\ \; & - & \mu ({IN}_{1}-{OUT}_{1},{IN}_{2}-{OUT}_{2},{IN}_{3}-{OUT}_{3},{IN}_{4}-{OUT}_{4})|= & \; & \\ \; & = & |\mu (5,1,6,7)-\mu (10,6,-5,9)|=|4.75-5|=0.25; & \; & \\ \Delta_{4}\mu {\left(IN+OUT\right)}_{2} & = & |\mu \left({IN}_{5}+{OUT}_{5}\right),{IN}_{6}+{OUT}_{6},{IN}_{7}+{OUT}_{7},{IN}_{8}+{OUT}_{8})- & \; & \\ \; & - & \mu \left({IN}_{1}+{OUT}_{1}\right),{IN}_{2}+{OUT}_{2},{IN}_{3}+{OUT}_{3},{IN}_{4}+{OUT}_{4})|= & \; & \\ \; & = & |\mu (21,15,16,27)-\mu (30,18,25,27)|=|19.75-25|= & \; & \\ \; & = & 5.25. & \; & \end{array}$$

**A Necessary Condition for Independence Theorem** **(NCIT):** If $X_{t}$, $X_{t-1}$, $Y_{t}$, $Y_{t-1}$ are discrete independent random variables, then, for any $\epsilon_{X}$ and $\epsilon_{Y}$,

$$P\left(D_{\epsilon_{X}}\left(X_{t}\right)=1,D_{\epsilon_{Y}}\left(Y_{t}\right)=1\right)=P\left(D_{\epsilon_{X}}\left(X_{t}\right)=1\right)\cdot P\left(D_{\epsilon_{Y}}\left(Y_{t}\right)=1\right).$$

**Proof.** Let us consider the probability $P(X_{t}-X_{t-1}=x,Y_{t}-Y_{t-1}=y)$, where x,y∈R are arbitrary numbers. Since $X_{t}$, $X_{t-1}$, $Y_{t}$, $Y_{t-1}$ are discrete and independent, this probability can be written as

$$P(X_{t}-X_{t-1}=x,Y_{t}-Y_{t-1}=y)$$

$$\begin{array}{cc} \; & =\sum_{i}\sum_{j}P\left(X_{t}=x-a_{i},X_{t-1}=a_{i},Y_{t}=y-b_{j},Y_{t-1}=b_{j}\right)\\ \; & =\sum_{i}\sum_{j}P\left(X_{t}=x-a_{i}\right)P\left(X_{t-1}=a_{i}\right)P\left(Y_{t}=y-b_{j}\right)P\left(Y_{t-1}=b_{j}\right)\\ \; & =\sum_{i}P\left(X_{t}=x-a_{i}\right)P\left(X_{t-1}=a_{i}\right)\cdot \left(\sum_{j}P\left(Y_{t}=y-b_{j}\right)P\left(Y_{t-1}=b_{j}\right)\right)\\ \; & =\left(\sum_{i}P\left(X_{t}=x-a_{i}\right)P\left(X_{t-1}=a_{i}\right)\right)\cdot P\left(Y_{t}-Y_{t-1}=y\right)\\ \; & =P\left(X_{t}-X_{t-1}=x\right)P\left(Y_{t}-Y_{t-1}=y\right). \end{array}$$

Thus, $X_{t}-X_{t-1}$ and $Y_{t}-Y_{t-1}$ are independent discrete variables, and, consequently, $|X_{t}-X_{t-1}|$ and $|Y_{t}-Y_{t-1}|$ are independent as well. Therefore, for any $\epsilon_{X}$ and $\epsilon_{Y}$ in R, we have

$$P(|X_{t}-X_{t-1}|\geq \epsilon_{X},|Y_{t}-Y_{t-1}|\geq \epsilon_{Y})=P(|X_{t}-X_{t-1}|\geq \epsilon_{X},|Y_{t}-Y_{t-1}\left|\geq \epsilon_{Y}\right),$$

if and only if

$$P\left(D_{\epsilon_{X}}\left(X_{t}\right)=1,D_{\epsilon_{Y}}\left(Y_{t}\right)=1\right)=P\left(D_{\epsilon_{X}}\left(X_{t}\right)=1\right)\cdot P\left(D_{\epsilon_{Y}}\left(Y_{t}\right)=1\right).$$

Thus, $\forall \epsilon_{X}\in \left(0,\epsilon_{X}^{*}\right),\;\epsilon_{Y}\in \left(0,\epsilon_{Y}^{*}\right)$,

$$P\left(D_{\epsilon_{X}}\left(X_{t}\right)=1,D_{\epsilon_{Y}}\left(Y_{t}\right)=1\right)=P\left(D_{\epsilon_{X}}\left(X_{t}\right)=1\right)\cdot P\left(D_{\epsilon_{Y}}\left(Y_{t}\right)=1\right).$$

□
