# Supplementary material for: Integration of Scales and Cameras in Nondisruptive Electronic Beehive Monitoring: On the Within-Day Relationship of Hive Weight and Traffic in Honeybee (Apis mellifera) Colonies in Langstroth Hives in Tucson, Arizona, USA
Source: Sensors (Basel). 2022 Jun 25;22(13):4824. doi: 10.3390/s22134824 (PMC9269415; doi:10.3390/s22134824)
Supplement: Supplementary file 1 [file sensors-22-04824-s001.zip › sensors-1751736-supplementary.pdf]

Article

Support Materials: Integration of Scales and Cameras in Nondisruptive Electronic Beehive Monitoring: On the Within-Day Relationship of Hive Weight and Traffic in Honeybee (Apis Mellifera) Colonies in Langstroth Hives in Tucson, Arizona, USA

Vladimir Kulyukin[1,*], Anastasiia Tkachenko[1], Kristoffer Price[1], William Meikle[2], and Milagra Weiss[2]

[1] Department of Computer Science, Utah State University, Logan, UT 84322, USA; anastasiia.tkachenko@usu.edu (A.T.); kristoffer.price@usu.edu (K.P.)

[2] Carl Hayden Bee Research Center, USDA-ARS, Tucson, AZ 85719, USA; william.meikle@usda.gov (W.M.); milagra.weiss@usda.gov (M.W.)

* Correspondence: vladimir.kulyukin@usu.edu

Citation: Kulyukin, V.; Tkachenko, A.; Price, K.; Meikle, W.; Weiss, M. Support Materials: Integration of Scales and Cameras in Nondisruptive Electronic Beehive Monitoring: On the Relationship of Hive Weight and Traffic in Honeybee (Apis Mellifera) Colonies in Langstroth Hives in Tucson, Arizona, USA. *Sensors* **2022**, *22*, 4824. https://doi.org/10.3390/s22134824

Received:
Accepted:
Published:

Publisher's Note: MDPI stays neutral with regard to jurisdictional claims in published maps and institutional affiliations.

Copyright: © 2022 by the authors. Submitted to *Sensors* for possible open access publication under the terms and conditions of the Creative Commons Attri- bution (CC BY) license (https://creativecommons.org/licenses/by/4.0/).

Introduction

Since we have described the BeePIV algorithm in our previous publications cited in the main text of the article, we have included three video sets that illustrate how BeePIV counts bee motions in videos with different levels of bee traffic. BeePIV converts frames from bee traffic videos to particle motion frames with uniform white background. BeePIV applies particle image velocimetry (PIV) to these motion frames to compute particle displacement vector fields, classifies individual displacement vectors as incoming, outgoing, and lateral, and uses vector counts to measure incoming, outgoing, and lateral bee traffic. The first video is an original raw video captured by our deployed BeePi electronic beehive monitoring system; the second video is the corresponding video that consists of the white background motion frames extracted from the first video; the third video is the video of displacement vectors extracted by PIV from each pair of consecutive white background frames from the second video.

1. Video Sets

Video Set 1

1. This video shows 10 BeePi monitors deployed on 10 double super hives at a research apiary the USDA-ARS Carl Hayden Bee Research Center in Tucson, AZ May 1 – August 15, 2021; these monitors include the two monitors used in the study from May 15 to October 25, 2020 in Logan, Utah, USA reported in our article; the cardboard silver boxes in the video protect the BeePi miniature cameras against the elements and indicate which hives in the apiary had BeePi monitors deployed on them.
2. This video shows the inside of a BeePi monitor deployed on a double super Langstroth hive at the same research apiary the USDA-ARS Carl Hayden Bee Research Center in Tucson, AZ May 1 – August 15, 2021; this monitor is one of the two monitors deployed at the site apiary in Logan, Utah, USA from May 15 to October 25, 2020.

Video Set 2

1. beepiv_vid_0001.mp4 – original bee traffic video;
2. beepiv_motion_particles_0001.mp4 – video made up of motion particle frames extracted from the original video beepiv_vid_0001.mp4;

3. beepiv_vector_fields_0001.mp4 – video made up of frames with displacement vectors computed by PIV from every pair of consecutive motion particle frames in the video beepiv_motion_particles_0001.mp4

Video Set 3

1. beepiv_vid_0002.mp4 – original bee traffic video;
2. beepiv_motion_particles_0002.mp4 – video made up of motion particle frames extracted from the original video beepiv_vid_0002.mp4;
3. beepiv_piv_vector_fields_0002.mp4 – video made up of frames with displacement vectors computed by PIV from every pair of consecutive motion particle frames in the video beepiv_motion_particles_0002.mp4.

Video Set 4

1. beepiv_vid_0003.mp4 – original bee traffic video;
2. beepiv_motion_particles_0003.mp4 – video made up of motion particle frames ex- 4
tracted from the original video beepiv_vid_0003.mp4;
3. beepiv_piv_vector_fields_0003.mp4 – video made up of frames with displacement vectors computed by PIV from every pair of consecutive motion particle frames in the second video beepiv_motion_particles_0003.mp4.

Datasets

- 4_17_az_bmc_with_weights_srt.csv
- 4_19_az_bmc_with_weights_srt.csv
- 4_41_az_bmc_with_weights_srt.csv
- 4_43_az_bmc_with_weights_srt.csv
- 4_47_az_bmc_with_weights_srt.csv
- 4_53_az_bmc_with_weights_srt.csv

Software

Software for correlation and χ^2 data analysis is available here.

Tables

1.1. Suprema ϵ_W^* and ϵ_T^*

Hive 19

Table S1. Suprema ϵ_W^* and ϵ_T^* for hive H19 and for 5 types of traffic, 5 variances and 5 means; IN – incoming traffic; OUT – outgoing traffic; TOT – total traffic; LAT – lateral traffic; IN - OUT (difference between IN and OUT), IN + OUT (sum of IN and OUT).

Lag	Exact Measurement						Variance					Mean				
(hours)	W	IN	OUT	TOT	IN-OUT	IN+OUT	IN	OUT	TOT	IN-OUT	IN+OUT	IN	OUT	TOT	IN-OUT	IN+OUT
1	0.491	7.682	7.838	8.531	6.295	8.456	10.668	10.963	12.354	9.618	12.173	6.452	6.296	7.144	4.909	7.07
2	0.602	8.349	8.46	9.176	6.878	9.099	10.688	10.609	12.261	9.015	12.033	6.38	6.27	7.096	4.799	7.02
3	0.851	8.724	8.846	9.555	7.263	9.476	11.137	11.201	12.713	8.684	12.549	6.361	6.239	7.07	4.778	6.991
4	1.053	9.006	9.111	9.786	7.531	9.727	10.87	10.936	12.443	8.439	12.281	6.338	6.233	7.014	4.758	6.954
5	1.25	9.215	9.313	9.997	7.613	9.938	10.651	10.718	12.225	8.199	12.062	6.317	6.219	7.001	4.617	6.942
6	1.417	9.365	9.456	10.148	7.694	10.09	10.516	10.574	12.08	8.26	11.916	6.278	6.187	6.97	4.516	6.912

Hive 41

Table S2. Suprema ϵ_W^* and ϵ_T^* for hive H41 and for 5 types of traffic, 5 variances and 5 means; IN – incoming traffic; OUT – outgoing traffic; TOT – total traffic; LAT – lateral traffic; IN - OUT (difference between IN and OUT), IN + OUT (sum of IN and OUT).

**Lag**		**Exact Measurement**					**Variance**					**Mean**				
(hours)	W	IN	OUT	TOT	IN-OUT	IN+OUT	IN	OUT	TOT	IN-OUT	IN+OUT	IN	OUT	TOT	IN-OUT	IN+OUT
1	0.198	8.165	7.964	8.89	6.912	8.763	13.048	12.409	14.38	10.549	14.14	6.576	6.917	7.583	5.525	7.454
2	0.247	8.757	8.548	9.477	7.482	9.351	12.586	11.975	13.928	9.919	13.681	6.279	6.613	7.25	5.403	7.134
3	0.263	9.034	8.732	9.67	7.753	9.577	12.588	11.962	13.931	9.922	13.687	6.223	6.549	7.183	5.268	7.092
4	0.309	9.302	9.001	9.939	8.075	9.837	12.592	11.968	13.936	9.937	13.691	6.229	6.53	7.167	5.302	7.065
5	0.367	9.45	9.218	10.153	8.145	10.034	12.142	11.766	13.595	9.326	13.349	6.329	6.454	7.157	5.149	7.038
6	0.404	9.573	9.341	10.281	8.186	10.157	12.613	11.999	13.961	9.946	13.716	6.367	6.395	7.103	5.019	6.979

Hive 43

Table S3. Suprema ϵ_W^* and ϵ_T^* for hive H43 and for 5 types of traffic, 5 variances and 5 means; IN – incoming traffic; OUT – outgoing traffic; TOT – total traffic; LAT – lateral traffic; IN - OUT (difference between IN and OUT), IN + OUT (sum of IN and OUT).

**Lag**		**Exact Measurement**					**Variance**					**Mean**				
(hours)	W	IN	OUT	TOT	IN-OUT	IN+OUT	IN	OUT	TOT	IN-OUT	IN+OUT	IN	OUT	TOT	IN-OUT	IN+OUT
1	0.265	7.721	7.499	8.414	6.303	8.285	11.566	11.609	13.314	9.058	12.971	6.113	6.335	7.028	4.916	6.899
2	0.31	8.408	8.3	9.191	6.771	9.049	11.461	11.231	13.018	8.743	12.731	6.221	6.329	7.111	4.691	6.969
3	0.345	8.704	8.609	9.491	7.009	9.351	11.199	10.954	12.779	8.664	12.434	6.124	6.219	7.007	4.524	6.866
4	0.369	8.978	8.874	9.759	7.185	9.62	11.075	10.806	12.603	8.414	12.325	6.101	6.205	6.986	4.424	6.848
5	0.445	9.15	9.044	9.926	7.347	9.79	11.088	11.091	12.756	8.291	12.476	6.048	6.154	6.93	4.352	6.794
6	0.483	9.291	9.135	10.023	7.434	9.903	10.832	10.681	12.398	8.106	12.116	5.957	6.113	6.845	4.307	6.725

Hive 47

Table S4. Suprema ϵ_W^* and ϵ_T^* for hive H47 and for 5 types of traffic, 5 variances and 5 means; IN – incoming traffic; OUT – outgoing traffic; TOT – total traffic; LAT – lateral traffic; IN - OUT (difference between IN and OUT), IN + OUT (sum of IN and OUT).

**Lag**		**Exact Measurement**					**Variance**					**Mean**				
(hours)	W	IN	OUT	TOT	IN-OUT	IN+OUT	IN	OUT	TOT	IN-OUT	IN+OUT	IN	OUT	TOT	IN-OUT	IN+OUT
1	1.128	7.589	7.391	8.297	5.938	8.188	11.782	11.197	13.096	9.089	12.894	6.005	6.203	6.911	4.551	6.802
2	1.063	8.016	7.809	8.72	6.475	8.611	11.271	10.773	12.627	8.541	12.418	5.73	5.937	6.64	4.396	6.532
3	1.092	8.299	8.109	9.018	6.778	8.902	11.13	10.789	12.565	8.384	12.346	5.624	5.814	6.519	4.293	6.417
4	0.962	8.37	8.169	9.115	6.795	8.968	11.061	10.729	12.481	8.219	12.286	5.602	5.597	6.343	4.307	6.195
5	1.03	8.519	8.134	9.151	7.378	9.038	10.925	10.573	12.358	8.487	12.138	5.804	5.337	6.403	4.631	6.291
6	0.927	8.348	8.171	9.103	6.603	8.957	10.953	10.601	12.386	8.124	12.169	5.648	5.208	6.252	4.525	6.145

Hive 53

Table S5. Suprema ϵ_W^* and ϵ_T^* for hive H53 and for 5 types of traffic, 5 variances and 5 means; IN – incoming traffic; OUT – outgoing traffic; TOT – total traffic; LAT – lateral traffic; IN - OUT (difference between IN and OUT), IN + OUT (sum of IN and OUT).

**Lag**		**Exact**	**Measurement**				**Variance**					**Mean**				
(hours)	W	IN	OUT	TOT	IN-OUT	IN+OUT	IN	OUT	TOT	IN-OUT	IN+OUT	IN	OUT	TOT	IN-OUT	IN+OUT
1	1.142	7.837	7.836	8.664	6.889	8.514	11.539	11.496	13.142	10.085	12.899	6.419	6.45	7.278	5.502	7.128
2	1.429	8.609	8.559	9.43	7.743	9.278	11.311	11.303	12.925	9.585	12.69	6.48	6.53	7.35	5.663	7.198
3	0.779	8.964	8.92	9.785	8.109	9.635	11.376	11.321	13.018	9.293	12.731	6.435	6.479	7.3	5.624	7.15
4	1.409	9.208	9.11	9.975	8.204	9.831	11.396	11.266	13.051	9.648	12.718	6.337	6.436	7.203	5.431	7.058
5	1.344	9.401	9.195	10.057	8.277	9.917	11.316	11.315	12.936	9.709	12.7	6.564	6.475	7.376	5.281	7.214
6	1.045	9.531	9.263	10.151	8.326	10.062	11.052	10.964	12.712	9.739	12.361	6.2	6.353	6.987	5.148	6.884

1.2. Maximal Difference between Joint and Marginal Probabilities.

Hive 17

Table S6. The maxima and argmaxima of the absolute difference between the joint probability and the product of marginal probabiliof variances of traffic measurements for different lags for hive H17; each entry has the following format: maximum difference, ϵ_W, ϵ_T.

**Lag (hours)**	σ^2(**IN**)	σ^2(**OUT**)	*maxD*, ϵ_W, ϵ_T σ^2(**TOT**)	σ^2(**IN-OUT**)	σ^2(**IN+OUT**)
1	(0.076,0.070,7.382)	(0.077,0.051,8.352)	(0.082,0.051,9.477)	(0.039,0.097,5.848)	(0.082,0.051,9.187)
2	(0.073,0.145,8.755)	(0.069,0.147,9.304)	(0.073,0.147,10.901)	(0.044,0.142,6.966)	(0.070,0.147,10.678)
3	(0.072,0.152,8.141)	(0.067,0.115,9.256)	(0.068,0.152,10.871)	(0.062,0.152,6.097)	(0.067,0.115,10.506)
4	(0.052,0.308,7.615)	(0.054,0.199,7.310)	(0.061,0.199,9.193)	(0.044,0.565,7.466)	(0.061,0.199,8.939)
5	(0.063,0.345,10.684)	(0.060,0.052,9.410)	(0.061,0.052,10.530)	(0.062,0.295,6.330)	(0.062,0.052,10.243)
6	(0.098,0.113,8.454)	(0.105,0.132,10.364)	(0.096,0.404,12.008)	(0.068,0.132,7.077)	(0.100,0.404,11.862)

Table S7. The maxima and argmaxima of the absolute difference between the joint probability and the product of marginal probabilities of traffic means for different lags for hive H17; each entry has the following format: maximum difference, ϵ_W, ϵ_T.

**Lag (hours)**	μ(**IN**)	μ(**OUT**)	*maxD*, ϵ_W, ϵ_T μ(**TOT**)	μ(**IN-OUT**)	μ(**IN+OUT**)
1	(0.063,0.059,4.840)	(0.076,0.051,4.159)	(0.071,0.051,4.952)	(0.032,0.077,2.468)	(0.069,0.051,4.996)
2	(0.055,0.219,4.707)	(0.058,0.360,3.892)	(0.057,0.145,5.769)	(0.045,0.284,2.648)	(0.055,0.145,5.646)
3	(0.046,0.115,4.795)	(0.044,0.226,4.264)	(0.042,0.228,5.011)	(0.040,0.049,2.405)	(0.042,0.214,4.409)
4	(0.068,0.066,4.354)	(0.065,0.602,4.694)	(0.062,0.602,5.524)	(0.056,0.087,3.068)	(0.061,0.602,5.488)
5	(0.060,0.080,5.022)	(0.047,0.345,3.818)	(0.055,0.080,5.844)	(0.080,0.574,1.902)	(0.055,0.080,5.749)
6	(0.087,0.152,4.768)	(0.082,0.214,4.704)	(0.087,0.214,5.548)	(0.050,0.353,2.163)	(0.092,0.214,5.449)

Hive 19

Table S8. The maxima and argmaxima of absolute differences between joint probability and product of marginal probabilities of exact traffic measurements and different lags for hive H19; each entry has format ($maxD$, ϵ_W, ϵ_T).

Lag (hours)			$maxD, \epsilon_W, \epsilon_T$		
	**IN**	**OUT**	**TOT**	**IN-OUT**	**IN+OUT**
1	(0.039,0.056,4.804)	(0.033,0.035,5.537)	(0.035,0.035,5.835)	(0.031,0.048,4.419)	(0.036,0.067,5.684)
2	(0.050,0.076,6.583)	(0.047,0.075,6.644)	(0.055,0.076,7.313)	(0.048,0.145,4.787)	(0.053,0.074,7.186)
3	(0.044,0.138,7.872)	(0.043,0.120,7.341)	(0.046,0.149,8.103)	(0.054,0.181,5.273)	(0.046,0.120,7.968)
4	(0.050,0.088,8.048)	(0.049,0.150,7.788)	(0.049,0.150,8.471)	(0.043,0.111,5.620)	(0.051,0.150,8.421)
5	(0.068,0.148,7.363)	(0.063,0.148,7.565)	(0.068,0.148,8.182)	(0.074,0.151,5.935)	(0.068,0.148,8.139)
6	(0.101,0.101,8.545)	(0.097,0.159,8.720)	(0.097,0.101,9.375)	(0.081,0.238,6.023)	(0.097,0.101,9.311)

Table S9. The maxima and argmaxima of absolute difference between joint probability and product of marginal probabilities of variances of traffic measurements for different lags for hive H19; each entry has the following format: (maximum difference, ϵ_W, ϵ_T).

Lag (hours)			$maxD, \epsilon_W, \epsilon_T$		
	σ^2**(IN)**	σ^2**(OUT)**	σ^2**(TOT)**	σ^2**(IN-OUT)**	σ^2**(IN+OUT)**
1	(0.049,0.044,6.188)	(0.032,0.044,6.907)	(0.043,0.044,7.660)	(0.028,0.044,6.348)	(0.038,0.044,8.521)
2	(0.037,0.078,7.054)	(0.045,0.072,6.684)	(0.031,0.078,8.215)	(0.050,0.126,6.401)	(0.033,0.072,7.821)
3	(0.039,0.213,8.241)	(0.028,0.102,7.841)	(0.043,0.213,9.662)	(0.034,0.289,6.166)	(0.041,0.213,9.412)
4	(0.034,0.169,7.501)	(0.046,0.200,7.983)	(0.041,0.169,9.208)	(0.052,0.158,6.836)	(0.041,0.169,9.088)
5	(0.072,0.237,7.882)	(0.075,0.200,7.824)	(0.069,0.237,9.169)	(0.070,0.200,6.477)	(0.069,0.237,9.167)
6	(0.067,0.156,7.677)	(0.065,0.198,8.036)	(0.056,0.198,9.423)	(0.074,0.241,7.104)	(0.061,0.198,9.175)

Table S10. The maxima and argmaxima of absolute difference between joint probability and product of marginal probabilities of traffic means for different lags for hive H19; each entry has the following format: (maximum difference, ϵ_W, ϵ_T).

Lag (hours)			$maxD, \epsilon_W, \epsilon_T$		
	μ**(IN)**	μ**(OUT)**	μ**(TOT)**	μ**(IN-OUT)**	μ**(IN+OUT)**
1	(0.033,0.034,4.065)	(0.035,0.049,3.589)	(0.037,0.034,4.430)	(0.032,0.049,2.798)	(0.036,0.059,4.242)
2	(0.043,0.072,4.402)	(0.038,0.078,4.452)	(0.047,0.072,5.180)	(0.048,0.102,3.311)	(0.049,0.072,5.054)
3	(0.036,0.060,3.562)	(0.035,0.051,3.681)	(0.039,0.051,4.383)	(0.045,0.179,2.819)	(0.040,0.051,4.335)
4	(0.046,0.147,3.803)	(0.041,0.147,3.802)	(0.046,0.147,4.769)	(0.043,0.158,3.331)	(0.046,0.147,4.451)
5	(0.047,0.200,5.496)	(0.044,0.200,5.659)	(0.047,0.200,6.231)	(0.070,0.150,2.909)	(0.047,0.200,6.109)
6	(0.083,0.184,5.336)	(0.075,0.184,5.011)	(0.080,0.241,6.203)	(0.076,0.340,2.845)	(0.081,0.198,6.083)

Hive 41

Table S11. The maxima and argmaxima of absolute differences between joint probability and product of marginal probabilities of exact traffic measurements and different lags for hive H41; each entry has format ($maxD$, ϵ_W, ϵ_T).

Lag (hours)			$maxD, \epsilon_W, \epsilon_T$		
	**IN**	**OUT**	**TOT**	**IN-OUT**	**IN+OUT**
1	(0.037,0.020,6.422)	(0.040,0.027,6.203)	(0.037,0.026,6.950)	(0.031,0.025,4.220)	(0.037,0.021,6.793)
2	(0.032,0.047,6.834)	(0.044,0.047,6.267)	(0.039,0.047,7.526)	(0.046,0.026,5.805)	(0.039,0.047,7.347)
3	(0.039,0.035,7.125)	(0.038,0.042,6.621)	(0.040,0.042,7.602)	(0.041,0.042,6.441)	(0.038,0.048,7.545)
4	(0.075,0.037,7.117)	(0.075,0.040,7.891)	(0.075,0.037,7.760)	(0.080,0.054,7.229)	(0.078,0.040,8.646)
5	(0.110,0.047,8.179)	(0.114,0.047,8.205)	(0.117,0.047,8.805)	(0.100,0.059,7.438)	(0.123,0.047,8.695)
6	(0.110,0.066,8.524)	(0.115,0.066,7.887)	(0.110,0.066,9.140)	(0.114,0.066,7.487)	(0.110,0.066,9.071)

Table S12. The maxima and argmaxima of absolute difference between joint probability and product of marginal probabilities of variances of traffic measurements for different lags for hive H41; each entry has the following format: (maximum difference, ϵ_W, ϵ_T).

Lag (hours)	$maxD, \epsilon_W, \epsilon_T$ σ^2(IN)	σ^2(OUT)	σ^2(TOT)	σ^2(IN-OUT)	σ^2(IN+OUT)
1	(0.038,0.020,8.742)	(0.027,0.040,8.810)	(0.034,0.040,10.497)	(0.032,0.036,6.962)	(0.036,0.040,9.615)
2	(0.039,0.037,9.691)	(0.045,0.037,8.263)	(0.043,0.035 10.585)	(0.040,0.035,6.546)	(0.043,0.035,9.577)
3	(0.044,0.032,8.182)	(0.036,0.032,8.732)	(0.036,0.039,11.702)	(0.077,0.039,6.747)	(0.036,0.039,11.497)
4	(0.041,0.043,8.940)	(0.067,0.037,8.976)	(0.052,0.037 10.592)	(0.064,0.053,7.453)	(0.052,0.037 10.268)
5	(0.076,0.048,9.714)	(0.087,0.048,9.177)	(0.084,0.037 10.876)	(0.050,0.081,7.927)	(0.076,0.048 10.813)
6	(0.072,0.053,9.964)	(0.088,0.036,9.119)	(0.081,0.036 10.890)	(0.054,0.069,8.156)	(0.084,0.053,11.110)

Table S13. The maxima and argmaxima of absolute difference between joint probability and product of marginal probabilities of traffic means for different lags for hive H41; each entry has the following format: (maximum difference, ϵ_W, ϵ_T).

Lag (hours)	$maxD, \epsilon_W, \epsilon_T$ μ(IN)	μ(OUT)	μ(TOT)	μ(IN-OUT)	μ(IN+OUT)
1	(0.033,0.026,4.735)	(0.039,0.020,4.980)	(0.032,0.026,5.460)	(0.029,0.050,2.818)	(0.035,0.026,5.442)
2	(0.045,0.074,4.458)	(0.041,0.025,4.166)	(0.031,0.047,5.075)	(0.036,0.020,3.674)	(0.031,0.047,5.208)
3	(0.036,0.016,4.916)	(0.040,0.024,4.650)	(0.041,0.055,6.105)	(0.045,0.018,4.214)	(0.039,0.055,5.958)
4	(0.058,0.053,4.983)	(0.075,0.037,5.289)	(0.077,0.053,5.948)	(0.072,0.053,4.401)	(0.074,0.053,6.146)
5	(0.082,0.070,5.127)	(0.107,0.044,5.099)	(0.090,0.044,6.012)	(0.108,0.059,4.325)	(0.096,0.044,5.912)
6	(0.102,0.085,5.157)	(0.110,0.065,5.180)	(0.108,0.085,6.179)	(0.111,0.069,4.266)	(0.112,0.085,6.072)

Hive 43

Table S14. The maxima and argmaxima of absolute differences between joint probability and product of marginal probabilities of exact traffic measurements and different lags for hive H43; each entry has format ($maxD$, ϵ_W, ϵ_T).

Lag (hours)	$maxD, \epsilon_W, \epsilon_T$ IN	OUT	TOT	IN-OUT	IN+OUT
1	(0.034,0.034,5.537)	(0.033,0.044,6.059)	(0.030,0.036,6.449)	(0.029,0.034,4.828)	(0.027,0.034,6.310)
2	(0.070,0.109,6.977)	(0.072,0.109,7.060)	(0.068,0.109,7.724)	(0.048,0.066,4.710)	(0.072,0.109,7.645)
3	(0.040,0.143,7.613)	(0.044,0.126,7.857)	(0.042,0.144,8.687)	(0.052,0.131,5.357)	(0.046,0.126,8.536)
4	(0.078,0.126,7.779)	(0.077,0.053,7.218)	(0.076,0.065,8.445)	(0.050,0.166,5.313)	(0.076,0.097,8.455)
5	(0.078,0.192,7.952)	(0.088,0.203,7.852)	(0.077,0.223,8.742)	(0.057,0.050,6.125)	(0.084,0.203,8.618)
6	(0.066,0.158,8.282)	(0.082,0.087,7.989)	(0.079,0.128,8.943)	(0.083,0.114,6.560)	(0.073,0.128,8.833)

Table S15. The maxima and argmaxima of absolute difference between joint probability and product of marginal probabilities of variances of traffic measurements for different lags for hive H43; each entry has the following format: (maximum difference, ϵ_W, ϵ_T).

Lag (hours)	$maxD, \epsilon_W, \epsilon_T$ σ^2(IN)	σ^2(OUT)	σ^2(TOT)	σ^2(IN-OUT)	σ^2(IN+OUT)
1	(0.036,0.019,9.252)	(0.028,0.016,8.823)	(0.038,0.019 10.651	0.032,0.061,6.159	(0.035,0.019 10.247)
2	(0.064,0.031,9.169)	(0.064,0.031,8.760)	(0.064,0.031 10.154	0.050,0.040,6.820	(0.071,0.031 10.440)
3	(0.050,0.059,9.743)	(0.039,0.135,7.887)	(0.044,0.055 10.990	0.044,0.048,5.805	(0.052,0.055 10.818)
4	(0.070,0.111,8.639)	(0.054,0.111,7.996)	(0.061,0.111,9.956	(0.038,0.063,6.478	(0.059,0.111,9.983)
5	(0.068,0.076,9.757)	(0.054,0.191,9.982)	(0.054,0.191,11.736	0.057,0.102,6.384	(0.057,0.076 10.479)
6	(0.083,0.058,9.641)	(0.073,0.058,8.438)	(0.076,0.087,11.282	0.049,0.053,6.080	(0.076,0.087,11.026)

Table S16. The maxima and argmaxima of absolute difference between joint probability and product of marginal probabilities of traffic means for different lags for hive H43; each entry has the following format: (maximum difference, ϵ_W, ϵ_T).

Lag (hours)			$maxD$, ϵ_W, ϵ_T		
	μ(IN)	μ(OUT)	μ(TOT)	μ(IN-OUT)	μ(IN+OUT)
1	(0.028,0.080,5.013)	(0.028,0.035,4.117)	(0.026,0.042,5.833)	(0.029,0.035,3.392)	(0.024,0.050,5.726)
2	(0.049,0.105,4.603)	(0.074,0.105,4.747)	(0.064,0.105,4.836)	(0.053,0.065,2.627)	(0.063,0.105,4.879)
3	(0.046,0.069,4.593)	(0.052,0.024,4.478)	(0.044,0.024,5.115)	(0.049,0.024,2.533)	(0.040,0.024,5.012)
4	(0.062,0.166,4.698)	(0.078,0.122,4.964)	(0.069,0.166,5.589)	(0.070,0.155,2.610)	(0.069,0.055,5.136)
5	(0.065,0.111,5.262)	(0.050,0.098,5.293)	(0.048,0.098,6.029)	(0.059,0.049,3.003)	(0.048,0.098,5.911)
6	(0.044,0.097,5.421)	(0.082,0.116,4.891)	(0.077,0.145,5.681)	(0.078,0.072,2.886)	(0.073,0.145,5.515)

Hive 47

Table S17. The maxima and argmaxima of absolute differences between joint probability and product of marginal probabilities of exact traffic measurements and different lags for hive H47; each entry has format ($maxD$, ϵ_W, ϵ_T).

Lag (hours)			$maxD$, ϵ_W, ϵ_T		
	IN	OUT	TOT	IN-OUT	IN+OUT
1	(0.031,0.012,4.771)	(0.038,0.012,4.317)	(0.036,0.012,5.231)	(0.030,0.012,4.190)	(0.036,0.012,5.293)
2	(0.034,0.028,5.638)	(0.028,0.025,4.466)	(0.030,0.028,6.293)	(0.037,0.028,5.075)	(0.031,0.028,6.066)
3	(0.037,0.037,6.645)	(0.038,0.039,6.314)	(0.044,0.039,7.211)	(0.046,0.046,4.898)	(0.041,0.039,7.109)
4	(0.051,0.042,6.014)	(0.043,0.042,5.924)	(0.042,0.042,6.466)	(0.056,0.032,5.268)	(0.045,0.042,6.363)
5	(0.118,0.042,6.779)	(0.111,0.042,6.410)	(0.117,0.042,7.691)	(0.140,0.042,5.908)	(0.117,0.042,7.388)
6	(0.055,0.042,5.737)	(0.054,0.041,5.537)	(0.055,0.041,6.507)	(0.057,0.042,5.182)	(0.054,0.041,6.413)

Table S18. The maxima and argmaxima of absolute difference between joint probability and product of marginal probabilities of variances of traffic measurements for different lags for hive H47; each entry has the following format: (maximum difference, ϵ_W, ϵ_T).

Lag (hours)			$maxD$, ϵ_W, ϵ_T		
	σ^2(IN)	σ^2(OUT)	σ^2(TOT)	σ^2(IN-OUT)	σ^2(IN+OUT)
1	(0.028,0.011,6.009)	(0.030,0.011,5.823)	(0.025,0.011,8.512)	(0.019,0.011,4.908)	(0.029,0.011,6.705)
2	(0.023,0.011,5.748)	(0.030,0.011,5.494)	(0.026,0.011,7.197)	(0.018,0.043,5.125)	(0.026,0.011,6.954)
3	(0.056,0.066,7.012)	(0.053,0.066,6.797)	(0.056,0.066,8.293)	(0.050,0.033,5.869)	(0.059,0.066,8.148)
4	(0.040,0.048,8.407)	(0.040,0.048,7.939)	(0.035,0.048,9.735)	(0.045,0.077,5.589)	(0.035,0.048,9.706)
5	(0.086,0.041,8.303)	(0.078,0.041,7.613)	(0.078,0.041,9.268)	(0.073,0.041,6.196)	(0.078,0.041,9.346)
6	(0.040,0.065,8.105)	(0.052,0.065,7.633)	(0.051,0.065,9.537)	(0.035,0.102,7.230)	(0.048,0.065,9.370)

Table S19. The maxima and argmaxima of absolute difference between joint probability and product of marginal probabilities of traffic means for different lags for hive H47; each entry has the following format: (maximum difference, ϵ_W, ϵ_T).

Lag (hours)			$maxD$, ϵ_W, ϵ_T		
	μ(IN)	μ(OUT)	μ(TOT)	μ(IN-OUT)	μ(IN+OUT)
1	(0.031,0.011,2.942)	(0.029,0.011,3.350)	(0.034,0.011,3.939)	(0.032,0.011,1.957)	(0.031,0.011,3.877)
2	(0.039,0.021,3.953)	(0.021,0.043,1.840)	(0.030,0.032,4.051)	(0.031,0.032,2.989)	(0.028,0.032,3.919)
3	(0.068,0.033,3.880)	(0.039,0.033,4.070)	(0.057,0.033,4.694)	(0.050,0.066,2.404)	(0.054,0.033,4.556)
4	(0.072,0.048,3.698)	(0.042,0.067,3.414)	(0.059,0.048,4.630)	(0.058,0.077,2.369)	(0.059,0.048,4.522)
5	(0.130,0.041,4.121)	(0.108,0.041,3.896)	(0.128,0.041,4.738)	(0.142,0.041,3.010)	(0.125,0.041,4.592)
6	(0.106,0.046,4.405)	(0.070,0.028,3.594)	(0.086,0.046,4.751)	(0.098,0.046,2.896)	(0.086,0.046,4.670)

Hive 53

Table S20. The maxima and argmaxima of absolute differences between joint probability and product of marginal probabilities of exact traffic measurements and different lags for hive H53; each entry has format ($maxD$, ϵ_W, ϵ_T).

Lag (hours)	$maxD, \epsilon_W, \epsilon_T$<br>IN	μ(OUT)	TOT	IN-OUT	IN+OUT
1	(0.026,0.034,3.526)	(0.026,0.039,2.996)	(0.024,0.038,6.194)	(0.021,0.016,4.127)	(0.024,0.034,4.804)
2	(0.034,0.064,4.522)	(0.031,0.064,4.317)	(0.041,0.064,4.942)	(0.036,0.068,3.912)	(0.038,0.064,4.673)
3	(0.042,0.200,7.106)	(0.047,0.200,5.652)	(0.040,0.332,8.791)	(0.042,0.200,4.331)	(0.041,0.332,8.694)
4	(0.074,0.232,7.853)	(0.073,0.176,7.933)	(0.069,0.176,8.929)	(0.049,0.232,4.700)	(0.066,0.232,8.587)
5	(0.085,0.257,7.891)	(0.088,0.331,8.251)	(0.088,0.331,9.145)	(0.048,0.394,6.001)	(0.088,0.331,9.002)
6	(0.103,0.168,8.097)	(0.095,0.130,5.979)	(0.103,0.168,8.739)	(0.077,0.092,4.942)	(0.100,0.168,8.652)

Table S21. The maxima and argmaxima of absolute difference between joint probability and product of marginal probabilities of variances of traffic measurements for different lags for hive H53; each entry has the following format: (maximum difference, ϵ_W, ϵ_T).

Lag (hours)	$maxD, \epsilon_W, \epsilon_T$<br>$\sigma^2$(IN)	σ^2(OUT)	σ^2(TOT)	σ^2(IN-OUT)	σ^2(IN+OUT)
1	(0.024,0.126,6.000)	(0.017,0.148,4.943)	(0.021,0.034,6.834)	(0.017,0.023,4.135)	(0.021,0.046,6.320)
2	(0.030,0.086,5.882)	(0.029,0.229,7.008)	(0.026,0.071,7.367)	(0.034,0.071,3.355)	(0.030,0.229,8.376)
3	(0.051,0.226,8.532)	(0.045,0.203,8.378)	(0.048,0.226 10.024)	(0.049,0.195,6.041)	(0.050,0.203,9.675)
4	(0.057,0.225,8.775)	(0.058,0.225,7.773)	(0.055,0.225,9.527)	(0.046,0.240,5.499)	(0.058,0.197,9.920)
5	(0.050,0.309,9.053)	(0.058,0.309,9.052)	(0.054,0.309 10.737)	(0.065,0.376,6.311)	(0.055,0.309 10.414)
6	(0.077,0.272,9.062)	(0.085,0.272,9.100)	(0.084,0.125,9.661)	(0.096,0.157,6.330)	(0.077,0.125,9.888)

Table S22. The maxima and argmaxima of absolute difference between joint probability and product of marginal probabilities of traffic means for different lags for hive H53; each entry has the following format: (maximum difference, ϵ_W, ϵ_T).

Lag (hours)	$maxD, \epsilon_W, \epsilon_T$<br>$\mu$(IN)	μ(OUT)	μ(TOT)	μ(IN-OUT)	μ(IN+OUT)
1	(0.021,0.034,1.541)	(0.025,0.034,2.322)	(0.021,0.046,4.876)	(0.018,0.160,2.366)	(0.020,0.046,4.063)
2	(0.041,0.071,3.175)	(0.048,0.071,3.657)	(0.043,0.071,3.969)	(0.031,0.057,1.812)	(0.041,0.071,4.319)
3	(0.035,0.327,5.277)	(0.038,0.327,5.442)	(0.037,0.327,6.205)	(0.034,0.296,1.294)	(0.037,0.327,6.078)
4	(0.056,0.056,3.612)	(0.058,0.310,4.891)	(0.055,0.268,6.122)	(0.045,0.240,2.607)	(0.052,0.310,5.011)
5	(0.070,0.309,5.185)	(0.080,0.309,5.634)	(0.072,0.309,6.048)	(0.047,0.215,1.637)	(0.074,0.309,6.204)
6	(0.081,0.366,5.084)	(0.080,0.272,4.701)	(0.087,0.366,6.009)	(0.063,0.272,2.574)	(0.085,0.366,5.851)

1.3. Marginal Probabilities at Argmaxima

Hive 17

Table S23. Marginal probabilities of weight and exact traffic measurements at $\widehat{\epsilon_W}$ and $\widehat{\epsilon_T}$, argmaxima of absolute differences between the joint probability and the product of the marginal probabilities, for different lags for hive H17; each entry has format $(P(D_{\widehat{\epsilon_W}}(W_t) = 1), P(D_{\widehat{\epsilon_T}}(T_t) = 1))$.

Lag (hours)	IN	OUT	TOT	IN-OUT	IN+OUT
1	(0.469, 0.645)	(0.294, 0.618)	(0.432, 0.645)	(0.592, 0.539)	(0.408, 0.645)
2	(0.285, 0.494)	(0.311, 0.549)	(0.302, 0.549)	(0.485, 0.306)	(0.289, 0.549)
3	(0.627, 0.476)	(0.705, 0.488)	(0.614, 0.464)	(0.596, 0.825)	(0.620, 0.464)
4	(0.478, 0.164)	(0.485, 0.164)	(0.463, 0.164)	(0.575, 0.791)	(0.463, 0.164)
5	(0.475, 0.802)	(0.505, 0.802)	(0.406, 0.386)	(0.703, 0.238)	(0.396, 0.426)
6	(0.643, 0.531)	(0.643, 0.653)	(0.633, 0.531)	(0.296, 0.316)	(0.622, 0.531)

Table S24. Marginal probabilities of weight and traffic variances at $\widehat{\epsilon_W}$ and $\widehat{\epsilon_T}$, argmaxima of absolute differences between the joint probability and the product of the marginal probabilities, for different lags for hive H17; each entry has format $(P(D_{\widehat{\epsilon_W}}(W_t) = 1), P(D_{\widehat{\epsilon_T}}(T_t) = 1))$.

Lag (hours)	σ^2(IN)	σ^2(OUT)	σ^2(TOT)	σ^2(IN-OUT)	σ^2(IN+OUT)
1	(0.469, 0.568)	(0.364, 0.645)	(0.384, 0.645)	(0.518, 0.471)	(0.397, 0.645)
2	(0.468, 0.553)	(0.404, 0.545)	(0.374, 0.545)	(0.298, 0.566)	(0.379, 0.545)
3	(0.645, 0.608)	(0.518, 0.651)	(0.482, 0.608)	(0.596, 0.602)	(0.536, 0.651)
4	(0.813, 0.321)	(0.821, 0.470)	(0.806, 0.470)	(0.254, 0.172)	(0.806, 0.470)
5	(0.228, 0.376)	(0.644, 0.861)	(0.653, 0.861)	(0.634, 0.426)	(0.663, 0.861)
6	(0.724, 0.653)	(0.480, 0.633)	(0.367, 0.378)	(0.490, 0.633)	(0.357, 0.378)

Table S25. Marginal probabilities of weight and traffic means at $\widehat{\epsilon_W}$ and $\widehat{\epsilon_T}$, argmaxima of absolute differences between the joint probability and the product of the marginal probabilities, for different lags for hive H17; each entry has format $(P(D_{\widehat{\epsilon_W}}(W_t) = 1), P(D_{\widehat{\epsilon_T}}(T_t) = 1))$.

Lag (hours)	μ(IN)	μ(OUT)	μ(TOT)	μ(IN-OUT)	μ(IN+OUT)
1	(0.292, 0.618)	(0.430, 0.645)	(0.423, 0.645)	(0.583, 0.539)	(0.397, 0.645)
2	(0.421, 0.396)	(0.647, 0.226)	(0.306, 0.549)	(0.506, 0.306)	(0.319, 0.549)
3	(0.458, 0.651)	(0.566, 0.476)	(0.584, 0.464)	(0.596, 0.831)	(0.717, 0.488)
4	(0.552, 0.769)	(0.440, 0.164)	(0.425, 0.164)	(0.328, 0.739)	(0.418, 0.164)
5	(0.505, 0.802)	(0.782, 0.376)	(0.475, 0.802)	(0.713, 0.238)	(0.475, 0.802)
6	(0.592, 0.612)	(0.571, 0.500)	(0.561, 0.500)	(0.663, 0.398)	(0.571, 0.500)

Hive 19

Table S26. Marginal probabilities of weight and exact traffic measurements at $\widehat{\epsilon_W}$ and $\widehat{\epsilon_T}$, argmaxima of absolute differences between the joint probability and the product of the marginal probabilities, for different lags for hive H19; each entry has format $(P(D_{\widehat{\epsilon_W}}(W_t) = 1), P(D_{\widehat{\epsilon_T}}(T_t) = 1))$.

Lag (hours)	IN	OUT	TOT	IN-OUT	IN+OUT
1	(0.651, 0.503)	(0.466, 0.688)	(0.571, 0.686)	(0.443, 0.583)	(0.608, 0.432)
2	(0.440, 0.625)	(0.458, 0.630)	(0.479, 0.625)	(0.497, 0.365)	(0.500, 0.633)
3	(0.218, 0.484)	(0.415, 0.545)	(0.396, 0.444)	(0.476, 0.367)	(0.429, 0.545)
4	(0.315, 0.653)	(0.438, 0.493)	(0.438, 0.493)	(0.461, 0.580)	(0.443, 0.493)
5	(0.588, 0.533)	(0.564, 0.533)	(0.588, 0.533)	(0.400, 0.527)	(0.588, 0.533)
6	(0.402, 0.689)	(0.378, 0.549)	(0.390, 0.689)	(0.518, 0.421)	(0.390, 0.689)

Table S27. Marginal probabilities of weight and traffic variances at $\widehat{\epsilon_W}$ and $\widehat{\epsilon_T}$, argmaxima of absolute differences between the joint probability and the product of the marginal probabilities, for different lags for hive H19; each entry has format $(P(D_{\widehat{\epsilon_W}}(W_t) = 1), P(D_{\widehat{\epsilon_T}}(T_t) = 1))$.

Lag (hours)	σ^2(IN)	σ^2(OUT)	σ^2(TOT)	σ^2(IN-OUT)	σ^2(IN+OUT)
1	(0.753, 0.614)	(0.637, 0.602)	(0.765, 0.579)	(0.458, 0.598)	(0.534, 0.579)
2	(0.654, 0.630)	(0.794, 0.630)	(0.753, 0.630)	(0.427, 0.393)	(0.768, 0.654)
3	(0.451, 0.298)	(0.582, 0.629)	(0.498, 0.298)	(0.502, 0.182)	(0.513, 0.298)
4	(0.721, 0.416)	(0.685, 0.352)	(0.685, 0.416)	(0.342, 0.457)	(0.680, 0.416)
5	(0.721, 0.461)	(0.715, 0.461)	(0.691, 0.388)	(0.467, 0.461)	(0.691, 0.388)
6	(0.720, 0.555)	(0.683, 0.482)	(0.689, 0.482)	(0.311, 0.415)	(0.683, 0.341)

Table S28. Marginal probabilities of weight and traffic means at $\widehat{\epsilon_W}$ and $\widehat{\epsilon_T}$, argmaxima of absolute differences between the joint probability and the product of the marginal probabilities, for different lags for hive H19; each entry has format $(P(D_{\widehat{\epsilon_W}}(W_t) = 1), P(D_{\widehat{\epsilon_T}}(T_t) = 1))$.

Lag (hours)	μ(IN)	μ(OUT)	μ(TOT)	μ(IN-OUT)	μ(IN+OUT)
1	(0.464, 0.688)	(0.641, 0.503)	(0.561, 0.686)	(0.450, 0.583)	(0.600, 0.432)
2	(0.464, 0.630)	(0.424, 0.625)	(0.469, 0.625)	(0.266, 0.495)	(0.471, 0.625)
3	(0.756, 0.767)	(0.764, 0.775)	(0.735, 0.775)	(0.367, 0.411)	(0.731, 0.775)
4	(0.685, 0.484)	(0.694, 0.484)	(0.658, 0.498)	(0.219, 0.457)	(0.694, 0.484)
5	(0.188, 0.448)	(0.606, 0.539)	(0.224, 0.455)	(0.455, 0.527)	(0.224, 0.455)
6	(0.268, 0.482)	(0.348, 0.482)	(0.250, 0.476)	(0.543, 0.268)	(0.250, 0.476)

Hive 41

Table S29. Marginal probabilities of weight and exact traffic measurements at $\widehat{\epsilon_W}$ and $\widehat{\epsilon_T}$, argmaxima of absolute differences between the joint probability and the product of the marginal probabilities, for different lags for hive H41; each entry has format $(P(D_{\widehat{\epsilon_W}}(W_t) = 1), P(D_{\widehat{\epsilon_T}}(T_t) = 1))$.

Lag (hours)	IN	OUT	TOT	IN-OUT	IN+OUT
1	(0.340, 0.412)	(0.318, 0.312)	(0.400, 0.318)	(0.701, 0.333)	(0.408, 0.402)
2	(0.533, 0.343)	(0.607, 0.343)	(0.525, 0.343)	(0.442, 0.512)	(0.562, 0.343)
3	(0.643, 0.503)	(0.696, 0.450)	(0.690, 0.450)	(0.427, 0.439)	(0.684, 0.380)
4	(0.796, 0.511)	(0.438, 0.496)	(0.796, 0.511)	(0.270, 0.460)	(0.445, 0.496)
5	(0.570, 0.626)	(0.430, 0.626)	(0.589, 0.626)	(0.327, 0.523)	(0.579, 0.626)
6	(0.470, 0.490)	(0.550, 0.500)	(0.470, 0.490)	(0.400, 0.490)	(0.470, 0.490)

Table S30. Marginal probabilities of weight and traffic variances at $\widehat{\epsilon_W}$ and $\widehat{\epsilon_T}$, argmaxima of absolute differences between the joint probability and the product of the marginal probabilities, for different lags for hive H41; each entry has format $(P(D_{\widehat{\epsilon_W}}(W_t) = 1), P(D_{\widehat{\epsilon_T}}(T_t) = 1))$.

Lag (hours)	σ^2(IN)	σ^2(OUT)	σ^2(TOT)	σ^2(IN-OUT)	σ^2(IN+OUT)
1	(0.502, 0.404)	(0.333, 0.190)	(0.378, 0.197)	(0.511, 0.233)	(0.545, 0.205)
2	(0.293, 0.351)	(0.649, 0.409)	(0.525, 0.351)	(0.678, 0.607)	(0.698, 0.409)
3	(0.883, 0.532)	(0.649, 0.450)	(0.269, 0.357)	(0.737, 0.503)	(0.287, 0.357)
4	(0.328, 0.672)	(0.642, 0.504)	(0.650, 0.504)	(0.606, 0.460)	(0.650, 0.504)
5	(0.664, 0.607)	(0.701, 0.626)	(0.729, 0.664)	(0.393, 0.495)	(0.729, 0.626)
6	(0.520, 0.550)	(0.690, 0.670)	(0.670, 0.670)	(0.260, 0.430)	(0.550, 0.550)

Table S31. Marginal probabilities of weight and traffic means at $\widehat{\epsilon_W}$ and $\widehat{\epsilon_T}$, argmaxima of absolute differences between the joint probability and the product of the marginal probabilities, for different lags for hive H41; each entry has format $(P(D_{\widehat{\epsilon_W}}(W_t) = 1), P(D_{\widehat{\epsilon_T}}(T_t) = 1))$.

Lag (hours)	μ(IN)	μ(OUT)	μ(TOT)	μ(IN-OUT)	μ(IN+OUT)
1	(0.308, 0.312)	(0.410, 0.318)	(0.397, 0.318)	(0.686, 0.333)	(0.389, 0.318)
2	(0.545, 0.202)	(0.707, 0.471)	(0.624, 0.343)	(0.455, 0.603)	(0.517, 0.343)
3	(0.222, 0.333)	(0.614, 0.620)	(0.292, 0.339)	(0.304, 0.673)	(0.310, 0.339)
4	(0.328, 0.460)	(0.438, 0.496)	(0.453, 0.460)	(0.248, 0.460)	(0.453, 0.460)
5	(0.589, 0.533)	(0.579, 0.626)	(0.617, 0.626)	(0.318, 0.514)	(0.495, 0.626)
6	(0.500, 0.350)	(0.490, 0.490)	(0.300, 0.350)	(0.380, 0.430)	(0.300, 0.350)

Hive 43

Table S32. Marginal probabilities of weight and exact traffic measurements at $\widehat{\epsilon_W}$ and $\widehat{\epsilon_T}$, argmaxima of absolute differences between the joint probability and the product of the marginal probabilities, for different lags for hive H43; each entry has format $(P(D_{\widehat{\epsilon_W}}(W_t) = 1), P(D_{\widehat{\epsilon_T}}(T_t) = 1))$.

Lag (hours)	IN	OUT	TOT	IN-OUT	IN+OUT
1	(0.582, 0.454)	(0.370, 0.373)	(0.538, 0.440)	(0.327, 0.457)	(0.555, 0.454)
2	(0.419, 0.310)	(0.338, 0.310)	(0.443, 0.310)	(0.567, 0.452)	(0.414, 0.310)
3	(0.340, 0.293)	(0.220, 0.347)	(0.207, 0.280)	(0.467, 0.327)	(0.213, 0.347)
4	(0.550, 0.358)	(0.750, 0.608)	(0.592, 0.533)	(0.658, 0.292)	(0.517, 0.450)
5	(0.467, 0.333)	(0.467, 0.311)	(0.444, 0.278)	(0.389, 0.711)	(0.444, 0.311)
6	(0.689, 0.356)	(0.800, 0.578)	(0.733, 0.422)	(0.344, 0.467)	(0.722, 0.422)

Table S33. Marginal probabilities of weight and traffic variances at $\widehat{\epsilon_W}$ and $\widehat{\epsilon_T}$, argmaxima of absolute differences between the joint probability and the product of the marginal probabilities, for different lags for hive H43; each entry has format $(P(D_{\widehat{\epsilon_W}}(W_t) = 1), P(D_{\widehat{\epsilon_T}}(T_t) = 1))$.

Lag (hours)	σ^2(IN)	σ^2(OUT)	σ^2(TOT)	σ^2(IN-OUT)	σ^2(IN+OUT)
1	(0.293, 0.639)	(0.346, 0.639)	(0.303, 0.639)	(0.550, 0.272)	(0.300, 0.639)
2	(0.433, 0.671)	(0.500, 0.681)	(0.552, 0.686)	(0.390, 0.590)	(0.467, 0.676)
3	(0.387, 0.647)	(0.827, 0.333)	(0.393, 0.647)	(0.687, 0.627)	(0.380, 0.647)
4	(0.600, 0.400)	(0.733, 0.392)	(0.650, 0.400)	(0.500, 0.542)	(0.567, 0.400)
5	(0.456, 0.522)	(0.689, 0.578)	(0.656, 0.522)	(0.511, 0.478)	(0.622, 0.522)
6	(0.611, 0.656)	(0.767, 0.656)	(0.389, 0.644)	(0.700, 0.711)	(0.367, 0.567)

Table S34. Marginal probabilities of weight and traffic means at $\widehat{\epsilon_W}$ and $\widehat{\epsilon_T}$, argmaxima of absolute differences between the joint probability and the product of the marginal probabilities, for different lags for hive H43; each entry has format $(P(D_{\widehat{\epsilon_W}}(W_t) = 1), P(D_{\widehat{\epsilon_T}}(T_t) = 1))$.

Lag (hours)	μ(IN)	μ(OUT)	μ(TOT)	μ(IN-OUT)	μ(IN+OUT)
1	(0.180, 0.159)	(0.579, 0.454)	(0.200, 0.368)	(0.325, 0.457)	(0.197, 0.368)
2	(0.448, 0.310)	(0.443, 0.310)	(0.700, 0.314)	(0.576, 0.452)	(0.657, 0.438)
3	(0.573, 0.500)	(0.607, 0.807)	(0.687, 0.807)	(0.613, 0.807)	(0.620, 0.807)
4	(0.533, 0.275)	(0.450, 0.358)	(0.517, 0.275)	(0.517, 0.458)	(0.692, 0.583)
5	(0.289, 0.433)	(0.511, 0.822)	(0.322, 0.456)	(0.433, 0.711)	(0.289, 0.456)
6	(0.622, 0.367)	(0.500, 0.356)	(0.544, 0.367)	(0.544, 0.611)	(0.578, 0.367)

Hive 47

Table S35. Marginal probabilities of weight and exact traffic measurements at $\widehat{\epsilon_W}$ and $\widehat{\epsilon_T}$, argmaxima of absolute differences between the joint probability and the product of the marginal probabilities, for different lags for hive H47; each entry has format $(P(D_{\widehat{\epsilon_W}}(W_t) = 1), P(D_{\widehat{\epsilon_T}}(T_t) = 1))$.

Lag (hours)	IN	OUT	TOT	IN-OUT	IN+OUT
1	(0.407, 0.646)	(0.429, 0.635)	(0.437, 0.645)	(0.293, 0.646)	(0.408, 0.645)
2	(0.484, 0.531)	(0.581, 0.615)	(0.472, 0.531)	(0.345, 0.531)	(0.491, 0.531)
3	(0.300, 0.574)	(0.252, 0.548)	(0.326, 0.548)	(0.557, 0.426)	(0.322, 0.548)
4	(0.723, 0.538)	(0.592, 0.533)	(0.761, 0.538)	(0.543, 0.658)	(0.755, 0.538)
5	(0.435, 0.572)	(0.435, 0.572)	(0.370, 0.572)	(0.384, 0.572)	(0.420, 0.572)
6	(0.717, 0.580)	(0.710, 0.587)	(0.696, 0.587)	(0.464, 0.580)	(0.710, 0.587)

Table S36. Marginal probabilities of weight and traffic variances at $\widehat{\epsilon_W}$ and $\widehat{\epsilon_T}$, argmaxima of absolute differences between the joint probability and the product of the marginal probabilities, for different lags for hive H47; each entry has format $(P(D_{\widehat{\epsilon_W}}(W_t) = 1), P(D_{\widehat{\epsilon_T}}(T_t) = 1))$.

Lag (hours)	σ^2(IN)	σ^2(OUT)	σ^2(TOT)	σ^2(IN-OUT)	σ^2(IN+OUT)
1	(0.459, 0.645)	(0.410, 0.646)	(0.443, 0.645)	(0.385, 0.588)	(0.512, 0.645)
2	(0.677, 0.553)	(0.590, 0.811)	(0.528, 0.615)	(0.478, 0.584)	(0.522, 0.615)
3	(0.665, 0.257)	(0.604, 0.257)	(0.665, 0.257)	(0.500, 0.426)	(0.683, 0.257)
4	(0.424, 0.446)	(0.342, 0.467)	(0.391, 0.467)	(0.723, 0.239)	(0.353, 0.467)
5	(0.391, 0.572)	(0.377, 0.572)	(0.507, 0.572)	(0.464, 0.435)	(0.377, 0.572)
6	(0.377, 0.703)	(0.442, 0.420)	(0.457, 0.420)	(0.514, 0.754)	(0.449, 0.420)

Table S37. Marginal probabilities of weight and traffic means at $\widehat{\epsilon_W}$ and $\widehat{\epsilon_T}$, argmaxima of absolute differences between the joint probability and the product of the marginal probabilities, for different lags for hive H47; each entry has format $(P(D_{\widehat{\epsilon_W}}(W_t) = 1), P(D_{\widehat{\epsilon_T}}(T_t) = 1))$.

Lag (hours)	μ(IN)	μ(OUT)	μ(TOT)	μ(IN-OUT)	μ(IN+OUT)
1	(0.408, 0.632)	(0.412, 0.646)	(0.407, 0.645)	(0.380, 0.646)	(0.408, 0.645)
2	(0.286, 0.621)	(0.457, 0.531)	(0.497, 0.531)	(0.360, 0.531)	(0.503, 0.543)
3	(0.352, 0.557)	(0.330, 0.578)	(0.370, 0.578)	(0.583, 0.426)	(0.365, 0.578)
4	(0.402, 0.495)	(0.685, 0.310)	(0.299, 0.630)	(0.255, 0.614)	(0.304, 0.630)
5	(0.341, 0.601)	(0.420, 0.572)	(0.362, 0.601)	(0.391, 0.572)	(0.370, 0.601)
6	(0.275, 0.522)	(0.420, 0.703)	(0.304, 0.522)	(0.326, 0.522)	(0.304, 0.522)

Hive 53

Table S38. Marginal probabilities of weight and exact traffic measurements at $\widehat{\epsilon_W}$ and $\widehat{\epsilon_T}$, argmaxima of absolute differences between the joint probability and the product of the marginal probabilities, for different lags for hive H53; each entry has format $(P(D_{\widehat{\epsilon_W}}(W_t) = 1), P(D_{\widehat{\epsilon_T}}(T_t) = 1))$.

Lag (hours)	IN	OUT	TOT	IN-OUT	IN+OUT
1	(0.771, 0.558)	(0.804, 0.505)	(0.417, 0.510)	(0.388, 0.795)	(0.674, 0.558)
2	(0.758, 0.528)	(0.783, 0.528)	(0.801, 0.528)	(0.575, 0.500)	(0.820, 0.528)
3	(0.408, 0.215)	(0.677, 0.215)	(0.148, 0.121)	(0.596, 0.215)	(0.143, 0.121)
4	(0.282, 0.207)	(0.236, 0.299)	(0.213, 0.299)	(0.569, 0.207)	(0.264, 0.207)
5	(0.360, 0.247)	(0.260, 0.173)	(0.260, 0.173)	(0.373, 0.140)	(0.260, 0.173)
6	(0.386, 0.346)	(0.701, 0.449)	(0.386, 0.346)	(0.646, 0.528)	(0.394, 0.346)

Table S39. Marginal probabilities of weight and traffic variances at $\widehat{\epsilon_W}$ and $\widehat{\epsilon_T}$, argmaxima of absolute differences between the joint probability and the product of the marginal probabilities, for different lags for hive H53; each entry has format $(P(D_{\widehat{\epsilon_W}}(W_t) = 1), P(D_{\widehat{\epsilon_T}}(T_t) = 1))$.

Lag (hours)	σ^2(IN)	σ^2(OUT)	σ^2(TOT)	σ^2(IN-OUT)	σ^2(IN+OUT)
1	(0.641, 0.164)	(0.612, 0.516)	(0.698, 0.506)	(0.705, 0.644)	(0.693, 0.516)
2	(0.733, 0.528)	(0.565, 0.143)	(0.798, 0.528)	(0.839, 0.466)	(0.547, 0.134)
3	(0.475, 0.215)	(0.444, 0.215)	(0.426, 0.215)	(0.457, 0.224)	(0.480, 0.215)
4	(0.443, 0.207)	(0.552, 0.207)	(0.546, 0.207)	(0.569, 0.241)	(0.471, 0.207)
5	(0.473, 0.173)	(0.360, 0.173)	(0.413, 0.173)	(0.433, 0.193)	(0.413, 0.173)
6	(0.402, 0.213)	(0.331, 0.213)	(0.567, 0.449)	(0.417, 0.378)	(0.504, 0.449)

Table S40. Marginal probabilities of weight and traffic means at $\widehat{\epsilon_W}$ and $\widehat{\epsilon_T}$, argmaxima of absolute differences between the joint probability and the product of the marginal probabilities, for different lags for hive H53; each entry has format $(P(D_{\widehat{\epsilon_W}}(W_t) = 1), P(D_{\widehat{\epsilon_T}}(T_t) = 1))$.

Lag (hours)	μ(IN)	μ(OUT)	μ(TOT)	μ(IN-OUT)	μ(IN+OUT)
1	(0.515, 0.516)	(0.752, 0.558)	(0.414, 0.510)	(0.492, 0.119)	(0.565, 0.516)
2	(0.686, 0.528)	(0.618, 0.503)	(0.677, 0.528)	(0.568, 0.500)	(0.599, 0.503)
3	(0.188, 0.121)	(0.695, 0.650)	(0.166, 0.121)	(0.628, 0.215)	(0.161, 0.121)
4	(0.667, 0.667)	(0.362, 0.149)	(0.707, 0.667)	(0.448, 0.201)	(0.155, 0.092)
5	(0.360, 0.173)	(0.193, 0.167)	(0.240, 0.167)	(0.680, 0.293)	(0.227, 0.167)
6	(0.323, 0.157)	(0.394, 0.268)	(0.291, 0.157)	(0.520, 0.213)	(0.307, 0.157)

1.4. Chi-Square Test

Hive 17

Table S41. Chi-square statistics and p-values of variances of traffic counts for different lags for hive H17; 15 p values smaller than 0.05 are bolded; IN – incoming traffic; OUT – outgoing traffic; TOT – total traffic.

Lag			*C and P-value*		
(hours)	σ^2(**IN**)	σ^2(**OUT**)	σ^2(**TOT**)	σ^2(**IN-OUT**)	σ^2(**IN+OUT**)
1	(96.732; **0.005**)	(121.868; **0.000**)	(105.168; **0.001**)	(73.183; 0.202)	(95.296; **0.007**)
2	(36.809; **0.002**)	(37.992; **0.046**)	(36.730;0.061)	(20.823; 0.702)	(47.695; **0.004**)
3	(32.448; **0.009**)	(26.468; **0.048**)	(30.374; **0.016**)	(29.709; **0.020**)	(30.114; **0.017**)
4	(22.658; 0.123)	(19.717; 0.233)	(23.097; 0.111)	(29.990; **0.018**)	(25.741; 0.058)
5	(9.367; 0.404)	(9.115; 0.427)	(9.367; 0.404)	(8.345;0.500)	(7.130;0.624)
6	(26.373; **0.002**)	(23.344; **0.005**)	(16.460;0.058)	(11.193;0.263)	(14.942;0.093)

Table S42. Chi-square statistics and p-values for means of traffic counts for different lags for hive H17; 5 *p* values smaller than 0.05 are bolded; IN – incoming traffic; OUT – outgoing traffic; TOT – total traffic.

Lag (hours)			*C and P-value*		
	μ(**IN**)	μ(**OUT**)	μ(**TOT**)	μ(**IN-OUT**)	μ(**IN+OUT**)
1	(83.189;0.054)	(96.639; **0.005**)	(91.180; **0.014**)	(61.817;0.554)	(98.072; **0.004**)
2	(36.114;0.070)	(37.128; 0.056)	(38.512; **0.041**)	(29.382;0.248)	(39.465; **0.033**)
3	(18.455;0.298)	(14.359; 0.572)	(12.466;0.711)	(26.156;0.052)	(13.990; 0.599)
4	(23.234; 0.108)	(20.985;0.179)	(21.014;0.178)	(14.625;0.552)	(20.985; 0.179)
5	(13.879; 0.127)	(5.873; 0.753)	(5.898; 0.750)	(13.991; 0.123)	(7.788; 0.556)
6	(14.145; 0.117)	(12.210; 0.202)	(10.415;0.318)	(7.982;0.536)	(10.415;0.318)

Hive 19

Table S43. The chi-square statistics and p-values for different lags: Exact counts.

Lag (hours)			*C and P-value*		
	**IN**	**OUT**	**TOT**	**IN-OUT**	**IN+OUT**
1	(128.795,0.297)	(105.722,0.837)	(105.151,0.847)	(131.219,0.248)	(118.849,0.538)
2	(64.961,0.063)	(43.219,0.706)	(58.667,0.162)	(53.186,0.316)	(62.000,0.101)
3	(32.533,0.634)	(26.533,0.875)	(34.668,0.532)	(39.962,0.298)	(33.601,0.583)
4	(35.823,0.074)	(32.707,0.139)	(30.806,0.196)	(20.053,0.744)	(31.040,0.188)
5	(30.606,0.015)	(30.000,0.018)	(29.394,0.021)	(33.263,0.007)	(29.697,0.020)
6	(64.681,0.000)	(55.660,0.000)	(58.912,0.000)	(28.550,0.027)	(53.250,0.000)

Table S44. The chi-square statistics and p-values for different lags: Variances.

Lag (hours)			*C and P-value*		
	σ^2(**IN**)	σ^2(**OUT**)	σ^2(**TOT**)	σ^2(**IN-OUT**)	σ^2(**IN+OUT**)
1	(146.699,0.056)	(118.707,0.542)	(161.578,0.008)	(107.567,0.804)	(139.935,0.115)
2	(53.667,0.300)	(69.000,0.031)	(60.000,0.135)	(55.000,0.258)	(52.000,0.358)
3	(51.725,0.043)	(26.717,0.870)	(40.489,0.279)	(26.042,0.889)	(38.734,0.347)
4	(24.813,0.473)	(40.841,0.024)	(21.094,0.687)	(45.442,0.007)	(26.514,0.381)
5	(28.485,0.028)	(26.061,0.053)	(17.879,0.331)	(17.273,0.368)	(18.485,0.296)
6	(16.447,0.422)	(9.829,0.875)	(16.447,0.422)	(24.757,0.074)	(14.348,0.573)

Table S45. The chi-square statistics and p-values for different lags: Means.

Lag (hours)			*C and P-value*		
	μ(**IN**)	μ(**OUT**)	μ(**TOT**)	μ(**IN-OUT**)	μ(**IN+OUT**)
1	(114.908,0.639)	(127.501,0.325)	(96.713,0.949)	(125.912,0.362)	(94.698,0.963)
2	(48.333,0.500)	(52.986,0.323)	(60.181,0.131)	(50.583,0.411)	(54.243,0.281)
3	(28.773,0.799)	(36.411,0.450)	(43.317,0.187)	(56.030,0.018)	(45.707,0.129)
4	(25.909,0.412)	(25.675,0.425)	(20.294,0.731)	(23.214,0.565)	(22.296,0.619)
5	(20.303,0.207)	(25.758,0.058)	(22.121,0.139)	(20.909,0.182)	(16.364,0.428)
6	(38.200,0.001)	(36.911,0.002)	(38.464,0.001)	(30.494,0.016)	(38.416,0.001)

Hive 41

Table S46. The chi-square statistics and p-values for different lags: Exact counts.

Lag (hours)	*C and P-value*				
	**IN**	**OUT**	**TOT**	**IN-OUT**	**IN+OUT**
1	(65.691,0.418)	(66.784,0.382)	(77.324,0.122)	(70.661,0.265)	(71.379,0.246)
2	(25.141,0.455)	(25.024,0.461)	(25.254,0.448)	(31.750,0.165)	(28.668,0.278)
3	(14.185,0.585)	(12.688,0.695)	(15.436,0.493)	(15.428,0.494)	(16.003,0.453)
4	(22.492,0.128)	(18.189,0.313)	(21.947,0.145)	(22.388,0.131)	(19.874,0.226)
5	(17.978,0.035)	(18.802,0.027)	(17.036,0.048)	(12.017,0.212)	(17.978,0.035)
6	(19.680,0.020)	(22.880,0.006)	(21.600,0.010)	(15.520,0.078)	(23.840,0.005)

Table S47. The chi-square statistics and p-values for different lags: Variances.

Lag (hours)	*C and P-value*				
	σ^2**(IN)**	σ^2**(OUT)**	σ^2**(TOT)**	σ^2**(IN-OUT)**	σ^2**(IN+OUT)**
1	(57.457,0.705)	(67.100,0.371)	(86.071,0.034)	(76.235,0.141)	(73.034,0.2065)
2	(25.099,0.457)	(33.598,0.117)	(32.913,0.133)	(28.642,0.279)	(29.334,0.250)
3	(5.703,0.991)	(7.467,0.963)	(7.778,0.955)	(26.763,0.044)	(6.605,0.980)
4	(14.003,0.599)	(15.585,0.482)	(14.081,0.593)	(21.781,0.150)	(12.928,0.678)
5	(9.765,0.370)	(9.091,0.429)	(8.248,0.509)	(4.327,0.889)	(12.735,0.175)
6	(32.800,0.000)	(16.160,0.064)	(21.600,0.010)	(9.440,0.398)	(20.640,0.014)

Table S48. The chi-square statistics and p-values for different lags: Means.

Lag (hours)	*C and P-value*				
	μ**(IN)**	μ**(OUT)**	μ**(TOT)**	μ**(IN-OUT)**	μ**(IN+OUT)**
1	(73.786,0.189)	(83.017,0.055)	(71.788,0.236)	(69.142,0.308)	(66.646,0.386)
2	(28.095,0.304)	(27.060,0.353)	(29.201,0.256)	(32.538,0.143)	(28.659,0.278)
3	(15.677,0.476)	(23.656,0.097)	(11.011,0.809)	(14.777,0.541)	(10.419,0.844)
4	(15.273,0.505)	(22.647,0.123)	(28.364,0.029)	(19.650,0.236)	(23.347,0.105)
5	(9.844,0.363)	(17.204,0.046)	(13.198,0.154)	(15.043,0.090)	(14.654,0.101)
6	(22.560,0.007)	(24.800,0.003)	(21.920,0.009)	(16.800,0.052)	(23.520,0.005)

Hive 43

Table S49. The chi-square statistics and p-values for different lags: Exact counts.

Lag (hours)	*C and P-value*				
	**IN**	**OUT**	**TOT**	**IN-OUT**	**IN+OUT**
1	(48.047,0.932)	(61.455,0.567)	(65.298,0.431)	(50.581,0.889)	(51.986,0.859)
2	(30.686,0.200)	(35.486,0.080)	(30.686,0.200)	(24.186,0.509)	(32.743,0.138)
3	(15.667,0.476)	(18.667,0.286)	(18.667,0.286)	(24.135,0.087)	(22.333,0.133)
4	(10.133,0.340)	(12.267,0.199)	(10.133,0.340)	(7.067,0.630)	(12.267,0.199)
5	(25.755,0.002)	(23.693,0.005)	(22.980,0.006)	(7.129,0.624)	(24.840,0.003)
6	(8.884,0.448)	(19.381,0.022)	(17.686,0.039)	(8.445,0.490)	(13.719,0.133)

Table S50. The chi-square statistics and p-values for different lags: Variances.

Lag (hours)			*C and P-value*		
	σ^2**(IN)**	σ^2**(OUT)**	σ^2**(TOT)**	σ^2**(IN-OUT)**	σ^2**(IN+OUT)**
1	(58.475,0.671)	(38.183,0.996)	(77.900,0.114)	(62.777,0.520)	(63.478,0.495)
2	(26.914,0.360)	(28.971,0.265)	(35.143,0.086)	(29.314,0.251)	(30.000,0.224)
3	(20.000,0.220)	(8.333,0.938)	(16.333,0.430)	(10.667,0.830)	(17.667,0.344)
4	(5.867,0.753)	(5.333,0.804)	(6.667,0.672)	(8.533,0.481)	(6.667,0.672)
5	(12.346,0.194)	(3.390,0.947)	(7.791,0.555)	(12.789,0.172)	(8.720,0.463)
6	(13.018,0.162)	(11.100,0.269)	(11.417,0.248)	(7.361,0.600)	(9.675,0.377)

Table S51. The chi-square statistics and p-values for different lags: Means.

Lag (hours)			*C and P-value*		
	μ**(IN)**	μ**(OUT)**	μ**(TOT)**	μ**(IN-OUT)**	μ**(IN+OUT)**
1	(67.658,0.353)	(50.880,0.883)	(84.134,0.047)	(56.331,0.741)	(63.402,0.498)
2	(20.743,0.707)	(28.629,0.280)	(25.543,0.432)	(20.552,0.717)	(25.200,0.451)
3	(11.667,0.767)	(20.000,0.220)	(15.000,0.525)	(23.333,0.105)	(20.000,0.220)
4	(12.000,0.213)	(15.733,0.073)	(11.467,0.245)	(11.733,0.229)	(13.067,0.160)
5	(9.814,0.366)	(13.483,0.142)	(9.919,0.357)	(3.627,0.934)	(7.113,0.625)
6	(13.936,0.125)	(21.715,0.010)	(20.816,0.013)	(14.892,0.094)	(20.816,0.013)

Hive 47

Table S52. The chi-square statistics and p-values for different lags: Exact counts.

Lag (hours)			*C and P-value*		
	**IN**	**OUT**	**TOT**	**IN-OUT**	**IN+OUT**
1	(95.983,0.595)	(107.149,0.294)	(115.597,0.136)	(111.366,0.206)	(106.490,0.310)
2	(39.533,0.831)	(44.797,0.644)	(55.680,0.238)	(28.133,0.993)	(56.763,0.208)
3	(20.442,0.723)	(18.424,0.824)	(25.654,0.426)	(14.508,0.952)	(26.577,0.377)
4	(43.697,0.012)	(26.640,0.374)	(31.007,0.189)	(35.745,0.076)	(24.418,0.495)
5	(46.836,0.000)	(31.911,0.010)	(35.837,0.003)	(57.617,0.000)	(33.383,0.007)
6	(14.876,0.534)	(13.701,0.621)	(16.224,0.437)	(13.424,0.642)	(22.281,0.134)

Table S53. The chi-square statistics and p-values for different lags: Variances.

Lag (hours)			*C and P-value*		
	σ^2**(IN)**	σ^2**(OUT)**	σ^2**(TOT)**	σ^2**(IN-OUT)**	σ^2**(IN+OUT)**
1	(90.206,0.748)	(112.505,0.185)	(96.832,0.571)	(107.958,0.276)	(100.316,0.472)
2	(57.579,0.187)	(52.802,0.329)	(39.374,0.835)	(25.579,0.998)	(42.986,0.714)
3	(39.033,0.037)	(31.117,0.185)	(40.260,0.027)	(26.020,0.407)	(39.670,0.032)
4	(39.799,0.031)	(31.509,0.173)	(34.375,0.100)	(27.386,0.337)	(32.790,0.136)
5	(35.460,0.003)	(24.206,0.085)	(29.476,0.021)	(17.682,0.343)	(23.139,0.110)
6	(28.337,0.029)	(21.169,0.172)	(28.698,0.026)	(18.413,0.300)	(26.675,0.045)

Table S54. The chi-square statistics and p-values for different lags: Means.

Lag (hours)	μ(IN)	μ(OUT)	*C and P-value* μ(TOT)	μ(IN-OUT)	μ(IN+OUT)
1	(117.743,0.109)	(100.402,0.470)	(115.018,0.145)	(118.036,0.105)	(98.683,0.518)
2	(47.298,0.542)	(44.417,0.659)	(51.272,0.385)	(38.034,0.872)	(53.367,0.310)
3	(30.676,0.200)	(20.254,0.733)	(27.596,0.327)	(26.317,0.391)	(27.447,0.334)
4	(29.613,0.239)	(39.749,0.031)	(31.699,0.167)	(50.199,0.002)	(28.851,0.270)
5	(36.818,0.002)	(40.444,0.001)	(47.975,0.000)	(62.394,0.000)	(42.830,0.000)
6	(41.763,0.000)	(23.122,0.111)	(34.501,0.005)	(43.701,0.000)	(30.293,0.017)

Hive 53

Table S55. The chi-square statistics and p-values for different lags: Exact counts.

Lag (hours)	IN	OUT	*C and P-value* TOT	IN-OUT	IN+OUT
1	(105.732,0.328)	(102.772,0.405)	(100.076,0.479)	(115.242,0.141)	(122.151,0.066)
2	(43.503,0.695)	(41.771,0.758)	(41.813,0.757)	(45.576,0.613)	(39.838,0.822)
3	(26.691,0.372)	(29.108,0.259)	(20.484,0.721)	(25.169,0.453)	(24.268,0.504)
4	(33.570,0.006)	(29.062,0.024)	(35.241,0.004)	(18.814,0.278)	(32.598,0.008)
5	(50.000,0.000)	(46.667,0.000)	(55.667,0.000)	(19.667,0.236)	(50.000,0.000)
6	(38.964,0.001)	(42.988,0.000)	(46.118,0.000)	(20.206,0.211)	(44.493,0.000)

Table S56. The chi-square statistics and p-values for different lags: Variances.

Lag (hours)	σ^2(IN)	σ^2(OUT)	*C and P-value* σ^2(TOT)	σ^2(IN-OUT)	σ^2(IN+OUT)
1	(102.076,0.424)	(88.807,0.781)	(101.429,0.441)	(101.343,0.444)	(98.820,0.515)
2	(80.981,0.003)	(69.065,0.031)	(84.989,0.001)	(54.022,0.289)	(65.960,0.053)
3	(20.223,0.735)	(29.705,0.236)	(32.543,0.143)	(32.955,0.132)	(28.642,0.279)
4	(43.624,0.000)	(33.589,0.006)	(37.308,0.002)	(22.505,0.128)	(37.057,0.002)
5	(26.333,0.050)	(33.667,0.006)	(31.333,0.012)	(32.000,0.010)	(29.333,0.022)
6	(35.319,0.004)	(47.075,0.000)	(41.069,0.001)	(31.066,0.013)	(38.285,0.001)

Table S57. The chi-square statistics and p-values for different lags: Means.

Lag (hours)	μ(IN)	μ(OUT)	*C and P-value* μ(TOT)	μ(IN-OUT)	μ(IN+OUT)
1	(105.633,0.331)	(110.540,0.221)	(106.478,0.310)	(102.273,0.418)	(129.839,0.024)
2	(44.726,0.647)	(57.830,0.181)	(48.674,0.486)	(35.383,0.928)	(43.768,0.685)
3	(25.445,0.438)	(24.076,0.515)	(23.476,0.550)	(30.482,0.207)	(28.688,0.277)
4	(30.518,0.015)	(31.499,0.012)	(33.400,0.007)	(27.679,0.035)	(34.080,0.005)
5	(45.667,0.000)	(51.000,0.000)	(44.000,0.000)	(16.333,0.430)	(39.667,0.001)
6	(36.298,0.003)	(40.588,0.001)	(37.397,0.002)	(19.619,0.238)	(39.835,0.001)

1.5. Correlation Coefficients

Hive 19

Table S58. Correlation coefficients (Pearson's) and corresponding p-values of Incoming, Outgoing, Total, Incoming-Outgoing, and Incoming+Outgoing traffic measurements for different lags: **Hive 19**.

Lag (in hours)	IN	OUT	TOT	IN-OUT	IN+OUT
1	(0.166,9.00e-06)	(0.108,4.13e-03)	(0.138,2.35e-04)	(0.214,8.89e-09)	(0.136,2.84e-04)
2	(0.203,2.14e-04)	(0.136,1.34e-02)	(0.170,1.93e-03)	(0.249,4.85e-06)	(0.168,2.17e-03)
3	(0.213,1.51e-03)	(0.142,3.53e-02)	(0.179,8.01e-03)	(0.274,4.06e-05)	(0.177,8.85e-03)
4	(0.221,4.44e-03)	(0.146,6.17e-02)	(0.185,1.78e-02)	(0.294,1.34e-04)	(0.182,1.95e-02)
5	(0.227,1.76e-02)	(0.142,1.42e-01)	(0.183,5.64e-02)	(0.325,5.53e-04)	(0.182,5.80e-02)
6	(0.216,2.48e-02)	(0.131,1.76e-01)	(0.175,7.04e-02)	(0.328,5.34e-04)	(0.172,7.59e-02)

Hive 41

Table S59. Correlation coefficients (Pearson's) and corresponding p-values of Incoming, Outgoing, Total, Incoming-Outgoing, and Incoming+Outgoing traffic measurements for different lags: **Hive 41**.

Lag (in hours)	IN	OUT	TOT	IN-OUT	IN+OUT
1	(0.265,2.04e-08	(0.281,2.36e-09)	(0.287,1.03e-09)	(0.171,3.28e-04)	(0.273,7.20e-09)
2	(0.323,2.37e-06)	(0.340,6.64e-07)	(0.345,4.42e-07)	(0.232,8.52e-04)	(0.331,1.29e-06)
3	(0.337,7.47e-05)	(0.354,2.92e-05)	(0.359,2.18e-05)	(0.242,5.06e-03)	(0.345,4.79e-05)
4	(0.366,2.25e-04)	(0.383,1.07e-04)	(0.390,7.94e-05)	(0.275,6.39e-03)	(0.374,1.58e-04)
5	(0.395,8.57e-04)	(0.420,3.65e-04)	(0.421,3.51e-04)	(0.281,2.01e-02)	(0.407,5.80e-04)
6	(0.425,6.50e-04)	(0.447,3.01e-04)	(0.453,2.45e-04)	(0.303,1.75e-02)	(0.436,4.51e-04)

Hive 43

Table S60. Correlation coefficients (Pearson's) and corresponding p-values of Incoming, Outgoing, Total, Incoming-Outgoing, and Incoming+Outgoing traffic measurements for different lags: **Hive 43**.

Lag (in hours)	IN	OUT	TOT	IN-OUT	IN+OUT
1	(-0.177,4.52e-04)	(-0.119,1.91e-02)	(-0.152,2.69e-03)	(-0.355,5.14e-13)	(-0.150,3.05e-03)
2	(-0.181,1.52e-02)	(-0.115,1.24e-01)	(-0.153,4.10e-02)	(-0.437,8.57e-10)	(-0.150,4.45e-02)
3	(-0.199,2.96e-02)	(-0.126,1.71e-01)	(-0.167,6.77e-02)	(-0.504,4.45e-09)	(-0.164,7.28e-02)
4	(-0.199,6.03e-02)	(-0.127,2.34e-01)	(-0.168,1.13e-01)	(-0.505,3.92e-07)	(-0.165,1.20e-01)
5	(-0.302,1.92e-02)	(-0.227,8.07e-02)	(-0.268,3.84e-02)	(-0.555,4.24e-06)	(-0.267,3.90e-02)
6	(-0.226,8.25e-02)	(-0.146,2.67e-01)	(-0.193,1.39e-01)	(-0.594,5.70e-07)	(-0.188,1.49e-01)

Hive 47

Table S61. Correlation coefficients (Pearson's) and corresponding p-values of Incoming, Outgoing, Total, Incoming-Outgoing, and Incoming+Outgoing traffic measurements for different lags: **Hive 47**.

Lag (in hours)	IN	OUT	Total	IN-OUT	IN+OUT
1	(0.005,9.08e-01)	(0.003,9.33e-01)	(0.007,8.70e-01)	(0.007,8.56e-01)	(0.004,9.18e-01)
2	(0.061,3.12e-01)	(0.065,2.82e-01)	(0.066,2.76e-01)	(0.045,4.60e-01)	(0.063,2.97e-01)
3	(0.072,3.31e-01)	(0.077,3.01e-01)	(0.076,3.03e-01)	(0.051,4.92e-01)	(0.074,3.17e-01)
4	(0.073,3.94e-01)	(0.075,3.85e-01)	(0.078,3.64e-01)	(0.059,4.89e-01)	(0.074,3.89e-01)
5	(-0.025,8.15e-01)	(-0.040,7.03e-01)	(-0.028,7.89e-01)	(0.021,8.42e-01)	(-0.032,7.65e-01)
6	(0.068,5.19e-01)	(0.063,5.50e-01)	(0.070,5.08e-01)	(0.069,5.11e-01)	(0.066,5.31e-01)

Hive 53

Table S62. Correlation coefficients (Pearson's) and corresponding p-values of Incoming, Outgoing, Total, Incoming-Outgoing, and Incoming+Outgoing traffic measurements for different lags: **Hive 53**.

Lag (in hours)	IN	OUT	TOT	IN-OUT	IN+OUT
1	(0.571,3.72e-51)	(0.593,5.65e-56)	(0.588,5.87e-55)	(0.191,3.82e-06)	(0.584,5.79e-54)
2	(0.609,7.97e-30)	(0.634,6.23e-33)	(0.628,3.60e-32)	(0.217,2.55e-04)	(0.624,1.44e-31)
3	(0.656,2.66e-23)	(0.687,3.68e-26)	(0.679,2.22e-25)	(0.232,1.86e-03)	(0.674,6.92e-25)
4	(0.667,2.53e-18)	(0.701,7.72e-21)	(0.693,3.61e-20)	(0.261,2.54e-03)	(0.686,1.21e-19)
5	(0.638,4.79e-12)	(0.676,7.56e-14)	(0.664,2.88e-13)	(0.250,1.50e-02)	(0.658,5.92e-13)
6	(0.713,5.82e-14)	(0.748,7.09e-16)	(0.739,2.23e-15)	(0.314,4.08e-03)	(0.731,6.15e-15)

Figures

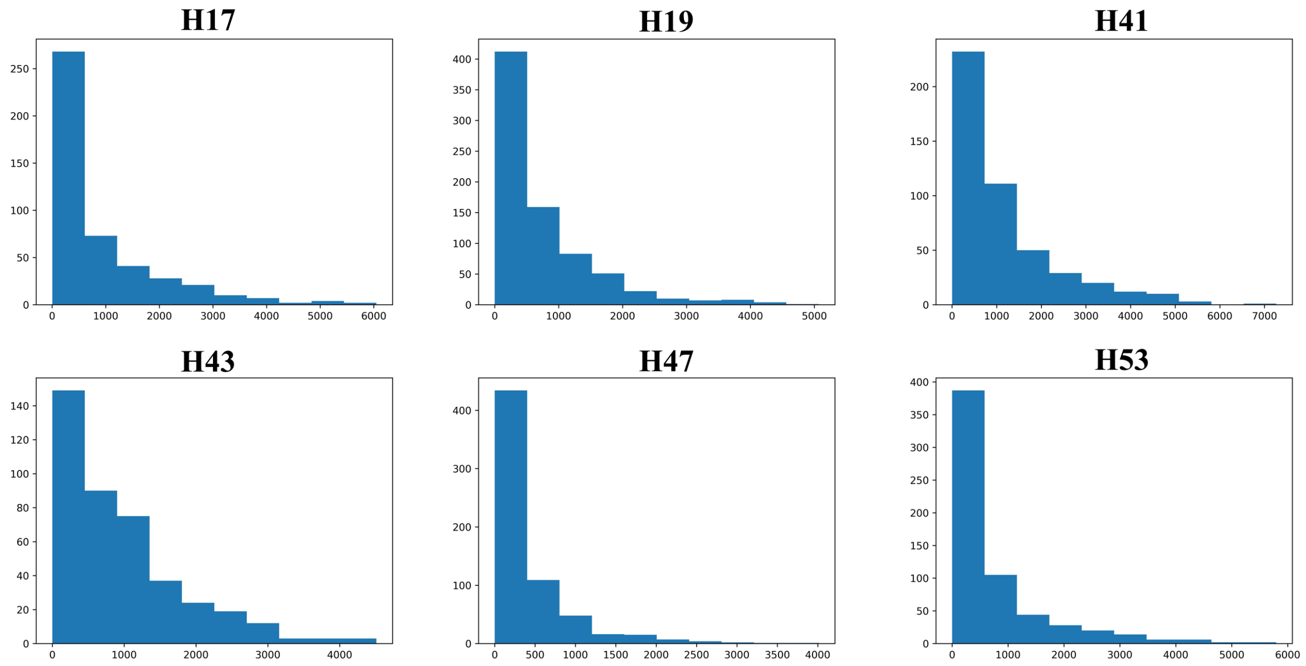

Figure S1. Histograms of the values of $\Delta_k T_t$ for $k = 4$ (lag of 1 hour) for all hives.

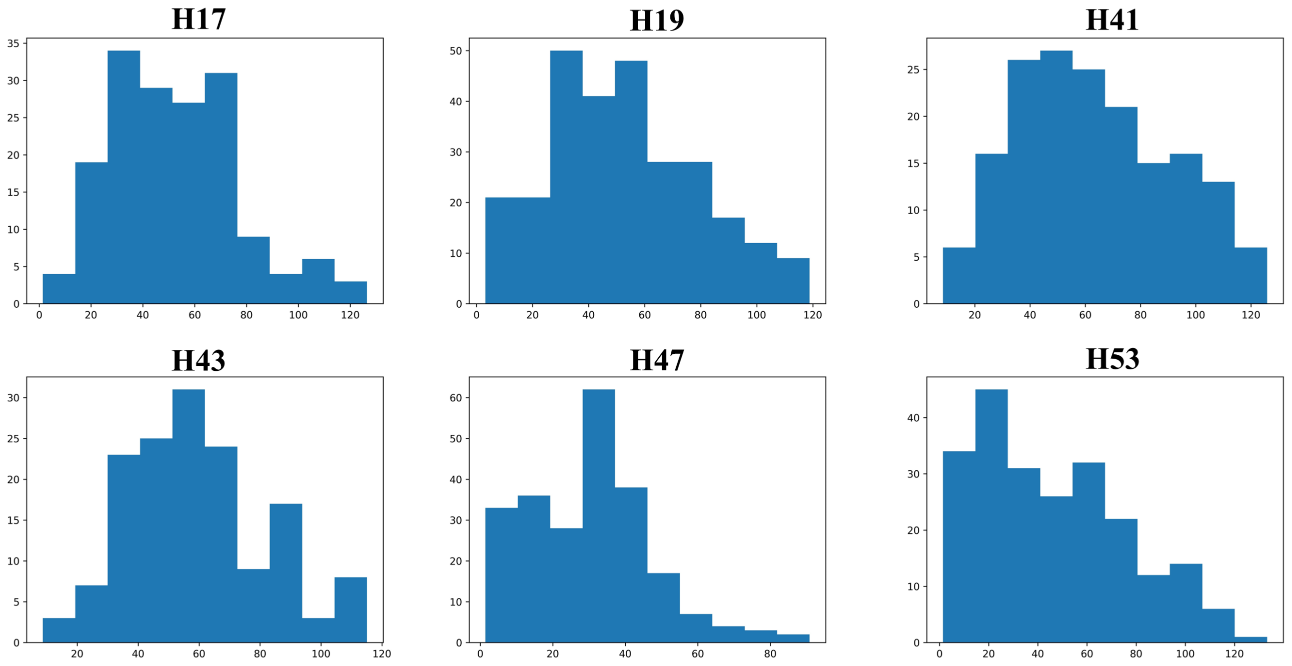

Figure S2. Histograms of the values of $\Delta_k T_t$ for $k = 12$ (lag of 3 hours) for all hives.

Below, we present autocorrelation plots for weight and total traffic for hives H19, H41, H43, H47, H53. The solid blue horizontal lines on each graph represent boundaries upon which if values fall outside of these, we have evidence against the null hypothesis that our correlation at lag L is equal to zero at the 5% level.

Hive 19

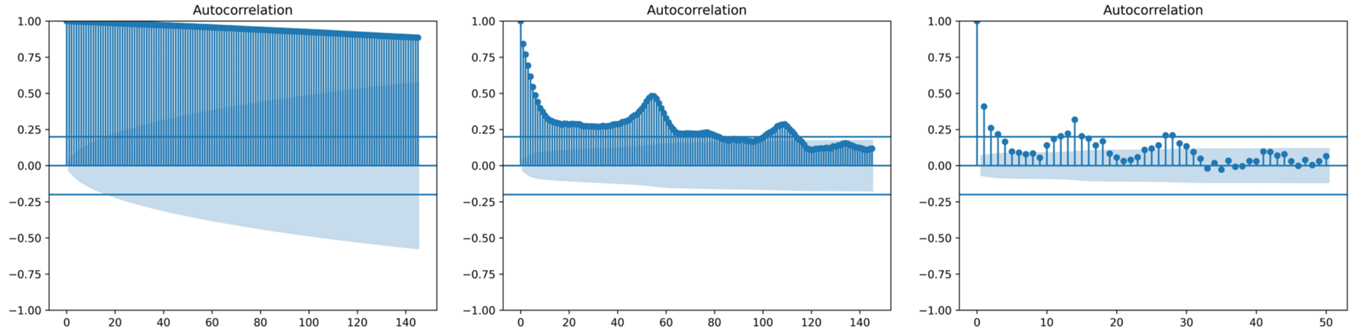

(a) Hive Weight – W **(b)** Total Traffic – TOT **(c)** Change of Weight – $\Delta_4 W_t$

Figure S3. Autocorrelation plots of weight **(a)**, total traffic (TOT) **(b)**, and change of weight over 1 hour **(c)** for hive H19 given by a standard python method *statsmodels.graphics.tsaplots*; the lags for **(a)**, **(b)** changes from 0 to 145, where value 145 approximately equals 2.5 periods as 54 is the full number of records per day; the lag for **(c)** runs from 0 to 50 ($\approx$ 3.5 periods) with a period of 12 (i.e., the number of data points per day for $k = 4$); semi-transparent solid blue regions represent confidence intervals for ACF values.

Hive 41

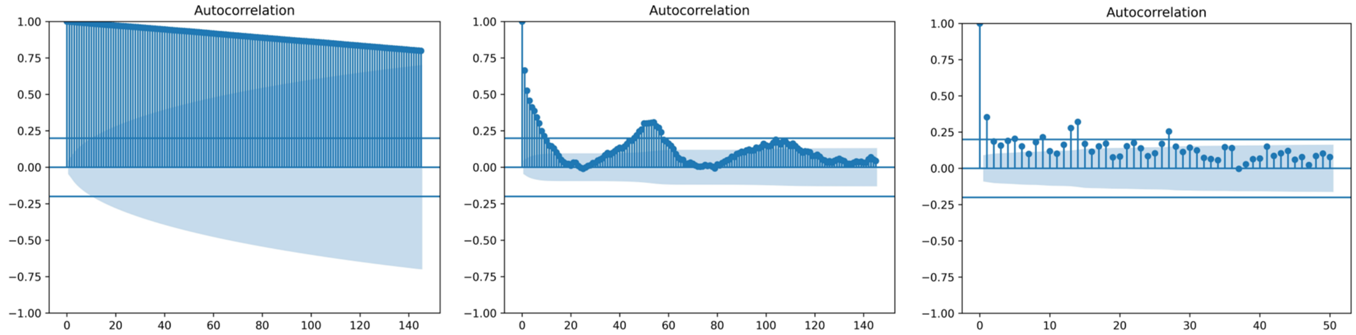

(a) Hive Weight – W **(b)** Total Traffic – TOT **(c)** Change of Weight – $\Delta_4 W_t$

Figure S4. Autocorrelation plots of weight **(a)**, total traffic (TOT) **(b)**, and change of weight over 1 hour **(c)** for hive H41 given by a standard python method *statsmodels.graphics.tsaplots*; the lags for **(a)**, **(b)** changes from 0 to 145, where value 145 approximately equals 2.5 periods as 54 is the full number of records per day; the lag for **(c)** runs from 0 to 50 ($\approx$ 3.5 periods) with a period of 12 (i.e., the number of data points per day for $k = 4$); semi-transparent solid blue regions represent confidence intervals for ACF values.

Hive 43

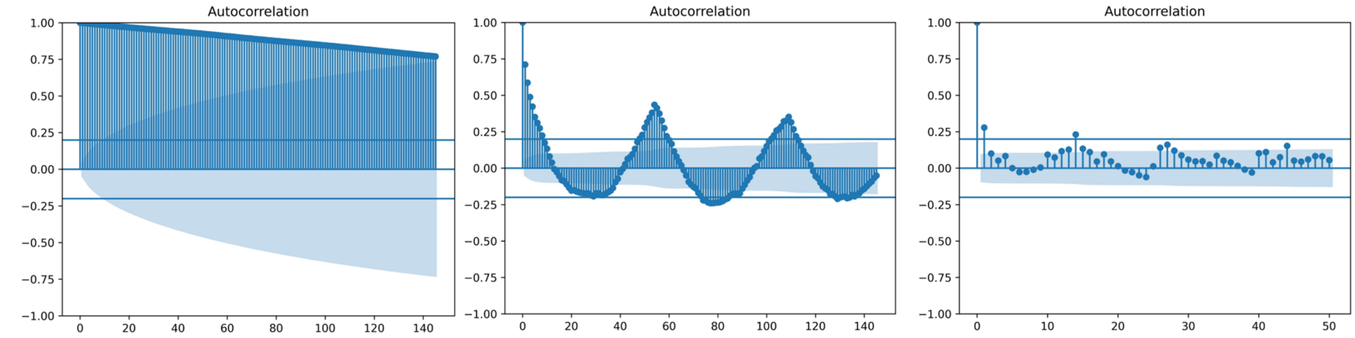

(a) Hive Weight – W **(b)** Total Traffic – TOT **(c)** Change of Weight – $\Delta_4 W_t$

Figure S5. Autocorrelation plots of weight **(a)**, total traffic (TOT) **(b)**, and change of weight over 1 hour **(c)** for hive H43 given by a standard python method *statsmodels.graphics.tsaplots*; the lags for **(a), (b)** changes from 0 to 145, where value 145 approximately equals 2.5 periods as 54 is the full number of records per day; the lag for **(c)** runs from 0 to 50 ($\approx$ 3.5 periods) with a period of 12 (i.e., the number of data points per day for $k = 4$); semi-transparent solid blue regions represent confidence intervals for ACF values.

Hive 47

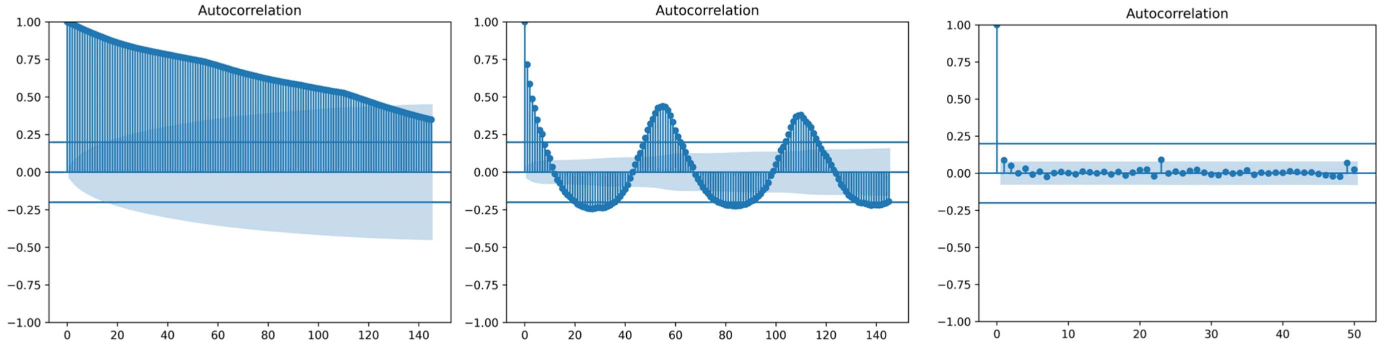

(a) Hive Weight – W **(b)** Total Traffic – TOT **(c)** Change of Weight – $\Delta_4 W_t$

Figure S6. Autocorrelation plots of weight **(a)**, total traffic (TOT) **(b)**, and change of weight over 1 hour **(c)** for hive H47 given by a standard python method *statsmodels.graphics.tsaplots*; the lags for **(a), (b)** changes from 0 to 145, where value 145 approximately equals 2.5 periods as 54 is the full number of records per day; the lag for **(c)** runs from 0 to 50 ($\approx$ 3.5 periods) with a period of 12 (i.e., the number of data points per day for $k = 4$); semi-transparent solid blue regions represent confidence intervals for ACF values.

Hive 53

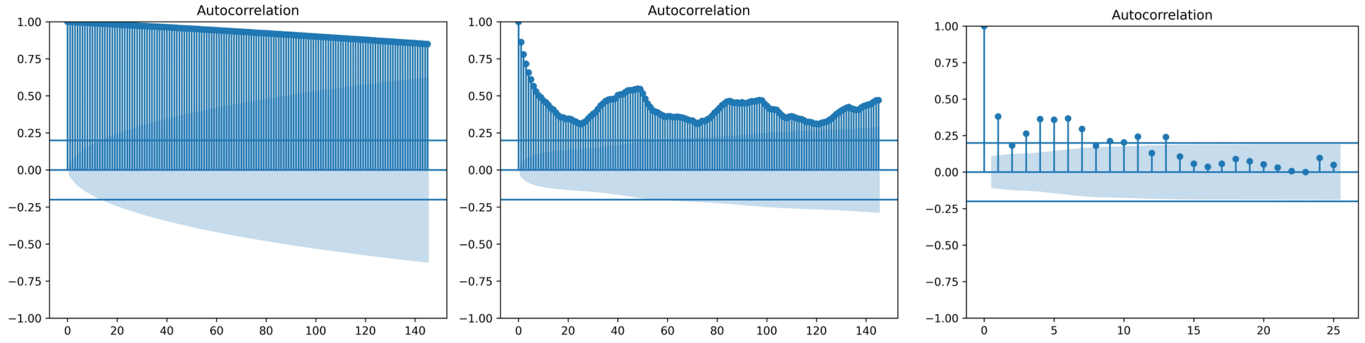

(a) Hive Weight – W **(b)** Total Traffic – TOT **(c)** Change of Weight – $\Delta_8 W_t$

Figure S7. Autocorrelation plots of weight **(a)**, total traffic (TOT) **(b)**, and change of weight over 1 hour **(c)** for hive H53 given by a standard python method *statsmodels.graphics.tsaplots*; the lags for **(a), (b)** changes from 0 to 145, where value 145 approximately equals 2.5 periods as 54 is the full number of records per day; the lag for **(c)** runs from 0 to 50 ($\approx$ 3.5 periods) with a period of 6 (i.e., the number of data points per day for $k = 8$); semi-transparent solid blue regions represent confidence intervals for ACF values.
